# A halotolerant growth promoting rhizobacteria triggers induced systemic resistance in plants and defends against fungal infection

**DOI:** 10.1038/s41598-019-40930-x

**Published:** 2019-03-11

**Authors:** Sandeep Sharma, Chen Chen, Sudhir Navathe, Ramesh Chand, Shree P. Pandey

**Affiliations:** 1 CSIR-Central Salt & Marine Chemicals Research Institute, Bhavnagar, India; 2 Academy of Scientific and Innovative Research, CSIR, Ghaziabad, India; 3 Jiangsu Key Laboratory of Crop Genetics and Physiology, Co-Innovation Center for Modern Production Technology of Grain Crops, Key Laboratory of Plant Functional Genomics of the Ministry of Education, Yangzhou University, Yangzhou, China; 4 Department of Mycology and Plant Pathology, Institute of Agricultural Sciences, Banaras Hindu University, Varanasi, India; 5 Department of Molecular Ecology, Max Planck Institute for Chemical Ecology, Jena, Germany

## Abstract

A halotolerant rhizobacteria, *Klebsiella* species (referred to MBE02), was identified that had a growth stimulation effect on peanut. To gain mechanistic insights into how molecular components were reprogrammed during the interaction of MBE02 and peanut roots, we performed deep RNA-sequencing. In total, 1260 genes were differentially expressed: 979 genes were up-regulated, whereas 281 were down-regulated by MBE02 treatment as compared to uninoculated controls. A large component of the differentially regulated genes were related to phytohormone signalling. This included activation of a significant proportion of genes involved in jasmonic acid, ethylene and pathogen-defense signalling, which indicated a role of MBE02 in modulating plant immunity. *In vivo* and *in vitro* pathogenesis assays demonstrated that MBE02 treatment indeed provide fitness benefits to peanut against *Aspergillus* infection under controlled as well as field environment. Further, MBE02 directly reduced the growth of a wide range of fungal pathogens including *Aspergillus*. We also identified possible molecular components involved in rhizobacteria-mediated plant protection. Our results show the potential of MBE02 as a biocontrol agent in preventing infection against several fungal phytopathogens.

## Introduction

Roots not only facilitate plants to uptake minerals and water from the soil but also serve as a major source of nutrients for microorganisms residing in the rhizosphere^1–3^. Root exudates are rich in organic compounds, which attract microbial population to colonize the plant roots^4^. Most of these microbes are neutral; however, many are pathogenic by nature and negatively affect the plant growth^1,5^. On the other hand, there are few microorganisms such as, mycorrhizal fungi, rhizobium and plant growth promoting rhizobacteria (PGPR), which produce beneficiary changes and stimulate plant growth via direct or indirect mechanisms^3,4,6–8^. One of these beneficiary changes include improved root architecture by increasing the number of root hairs and lateral root numbers. This may be correlated with the signalling of phytohormones, mainly auxin^2^. This enlargement in root surface facilitates plants to uptake more water and nutrient, and in turn leads to improved growth. In addition, colonization of plant roots by few PGPR strains trigger an induced systemic resistance (ISR) response that functions systemically throughout the plant and is often effective against a broad spectrum of plant pathogens^5,9,10^. In contrast to the costly defenses that often, upon activation, restricts the plant growth, ISR is associated with a defence priming phenomenon, which simultaneously stimulates plant growth and immunity^5,6,11,12^. These beneficial microbes could be an ideal choice for developing them into biocontrol agents that enhances disease resistance in crop plants.

Over the past few years, our understandings about molecular mechanisms of PGPR mediated growth promotion and disease resistance in plants has improved significantly as several genetic determinants that regulate these processes have been identified^13^. An auxin-dependent developmental change in root architecture has been identified as a key mechanism of plant growth stimulation by PGPR^3,7,14^. On the other hand, ISR is primarily governed by the components involved in ethylene (ET) and jasmonic acid (JA) signalling^5^. By using large-scale transcriptome analysis and reverse genetics approaches, several components, such as *MYB72*, β-glucosidase U42 (*BGLU42*) and *MYC2*, involved in rhizobacteria-mediated ISR have been identified^15–17^. However, considering complex interactive mechanisms of ISR, it is plausible that there are many yet-to-be-identified signalling components that might play important role(s) in this process. Moreover, most of this information has come from the model plant, *Arabidopsis*, and such mechanisms have not been explored in economically important crop species, and their role is yet not completely established in the agro-ecological habitats where crops are growing.

Peanut (*Arachis hypogaea* L.) is an important leguminous cash crop, which is widely grown in tropical and sub-tropical region. It serves as a good source of dietary protein, fats, vitamins, minerals and micronutrients^18^. Peanut production is severely reduced by attack of *Aspergillus* species, which are fungal pathogens^19,20^. *Aspergillus* species produces aflatoxins which are carcinogenic to animals including humans. Peanut has been considered as one of the most susceptible crops for *Aspergillus* and serve as the main source of mycotoxin exposure to humans and other animals^21,22^. *Aspergillus* causes seed rots, moulding of seeds, pre- and post-emergence damping off, and reductions in the seed viability and seedling growth in peanuts^21^. Soil is the main source of *Aspergillus* inoculum and, since pods grow below ground, they come in direct contact to *Aspergillus* population in the soil. Consumption of peanut that contains mycotoxin can cause liver cancer, growth retardation in children’s and may also lead to AIDS by suppressing the immune response^23^. Therefore, it is important to prevent the *Aspergillus* infection in peanut not only to reduce the yield losses but, also, to minimize the health risk. Application of rhizobacteria is an efficient and eco-friendly approach for simultaneous management of various plant diseases^5,24,25^. Few rhizobacteria have been shown to reduce the growth of various *Aspergillus* species^22,26–28^. However, underlying mechanisms is not yet known in detail.

Previously, we identified five PGPR’s from the roots of a halotolerant plant species, *Arthrocnemum indicum*, and characterized them^7^. We found that these bacteria, including the *Klebsiella spp* (referred to MBE02), has sticking growth promoting effects on peanut under non-stress conditions^7^. But the molecular machinery of the plant that is altered due to *Klebsiella* colonization, which might promote growth is unknown. Here, we broaden our understanding about the beneficial effects of colonization of peanut by *Klebsiella spp* (referred to MBE02) under field condition. We develop insights into the molecular response of plants as a consequence of MBE02 colonization with the help of whole transcriptome analysis (by deep RNA-sequencing). RNA-Seq analysis of roots of colonized peanut seedlings not only unravelled the molecular components involved in the growth stimulation, but also demonstrated elicitation of ISR/defense responses indicating MBE02’s plausible role in disease resistance. Thus, we investigate this hypothesis and explored potential of MBE02 as an effective biocontrol agent as we could show that MBE02 prevents fungal infections in the peanut.

## Results

### MBE02 treatment improves peanut growth under field conditions

**Figure 1:**
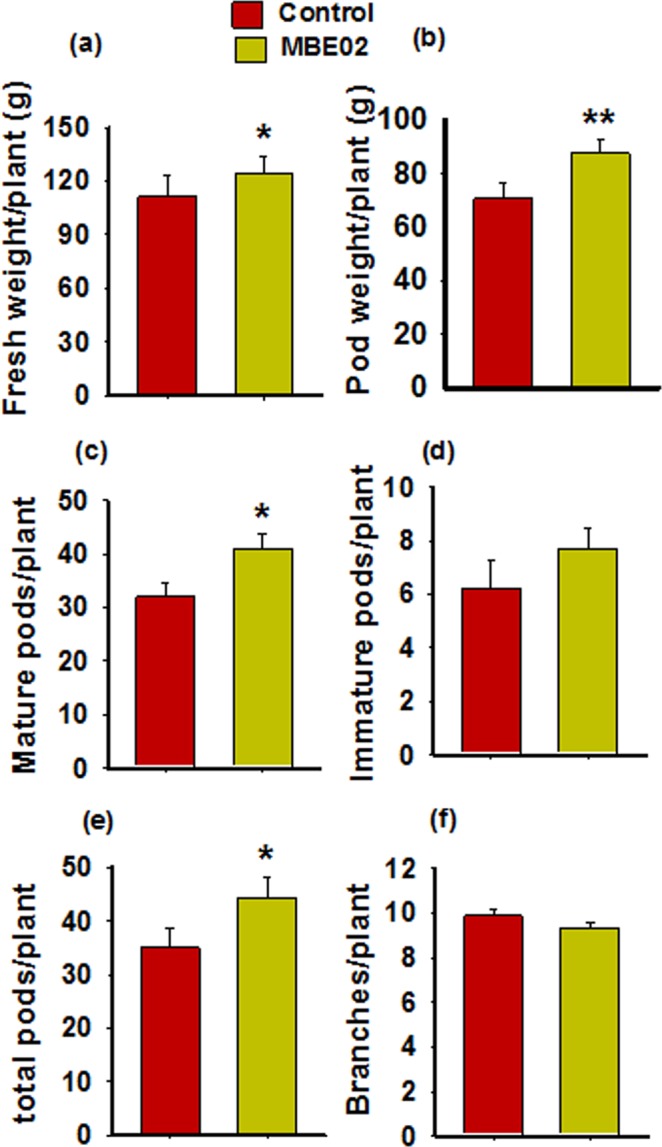
The rhizobacteria strain MBE02 improves peanut growth and yield under field condition. Yield parameters such as plant biomass (**a**), pod yield (**b**), mature pods (**c**), immature pods (**d**), total pods (**e**) and branches of MBE02-inoculated and non-inoculated plant were determined. Data are means ± SE (n = 30). Asterisks shows statistical difference calculated by t test. *Significance at p < 0.05 and **significance at 0.01.

Effect of MBE02 on the performance of peanut was evaluated in a field trial in the year 2016 at field station, Bhavnagar^7^. We have now analyzed various yield parameters such as fresh weight, pod weight, pod numbers and branches (Fig. 1). Treatment with the beneficiary rhizobacteria, MBE02, resulted in significantly higher fresh weight (11.6% per plant), pod weight (23.6% per plant), number of mature pods (28% per plant), and total number of mature pods (26.4% per plant) of peanut as compared to non-inoculated controls under unstressed condition (Fig. 1; n = 25-30, t test P < 0.05). No difference in immature pods (Fig. 1d) and branches per plant (Fig. 1f) were observed in MBE02 than the control.

### Gene expression analysis of MBE02 treated peanut roots

In order to gain insights into molecular response of the host to MBE02 inoculation, global reprograming in transcriptome of the peanut roots was studied. A total of 131 million clean reads were generated that resulted into 313409 transcripts, 183427 unigenes and 128224 coding sequences (CDS). As the reference genome of the cultivated peanut is not available, we relied on the progenitor genomes for transcriptome assembly and annotation. We adapted a two-pronged, complementary approach for assembly and annotation of the genes of the transcriptome of the cultivated peanut (*A. hypogaea*). On one hand, we performed genome-guided assembly of the transcriptome by using the genomes of progenitor A (*A. duranensis*) and B (*A. ipaensis*). As a consequence, a total of 27969 (21.8%) assembled sequences of cultivated peanut matched to the transcripts annotated to the reference A genome (*A. duranensis*) whereas, 46078 (35.9%) assembled sequence matched to the transcripts of the B genome (*A. ipaensis*). On the other hand, we performed a BLAST analysis against the non-redundant protein (Nr) database of NCBI. A total of 120759 transcripts had a significant match in the database, of which, 48338 uniquely matched to transcripts of A genome, whereas 64428 matched to B genome, and 4112 were common to both (Fig. S1 and Table S1). Additionally, 612 CDS were annotated to *A. hypogaea*. (Supplementary Fig. S1 and Table S1). A total of 36 and 38 gene ontology (GO) terms were assigned to 845 and 777 CDS from control and MBE02 treatment, respectively (Supplementary Fig. S2). In addition, 8156 genes of control and 8042 genes of bacteria treatment were classified into 24 functional categories by KAAS (Supplementary Table S2).

**Figure 2:**
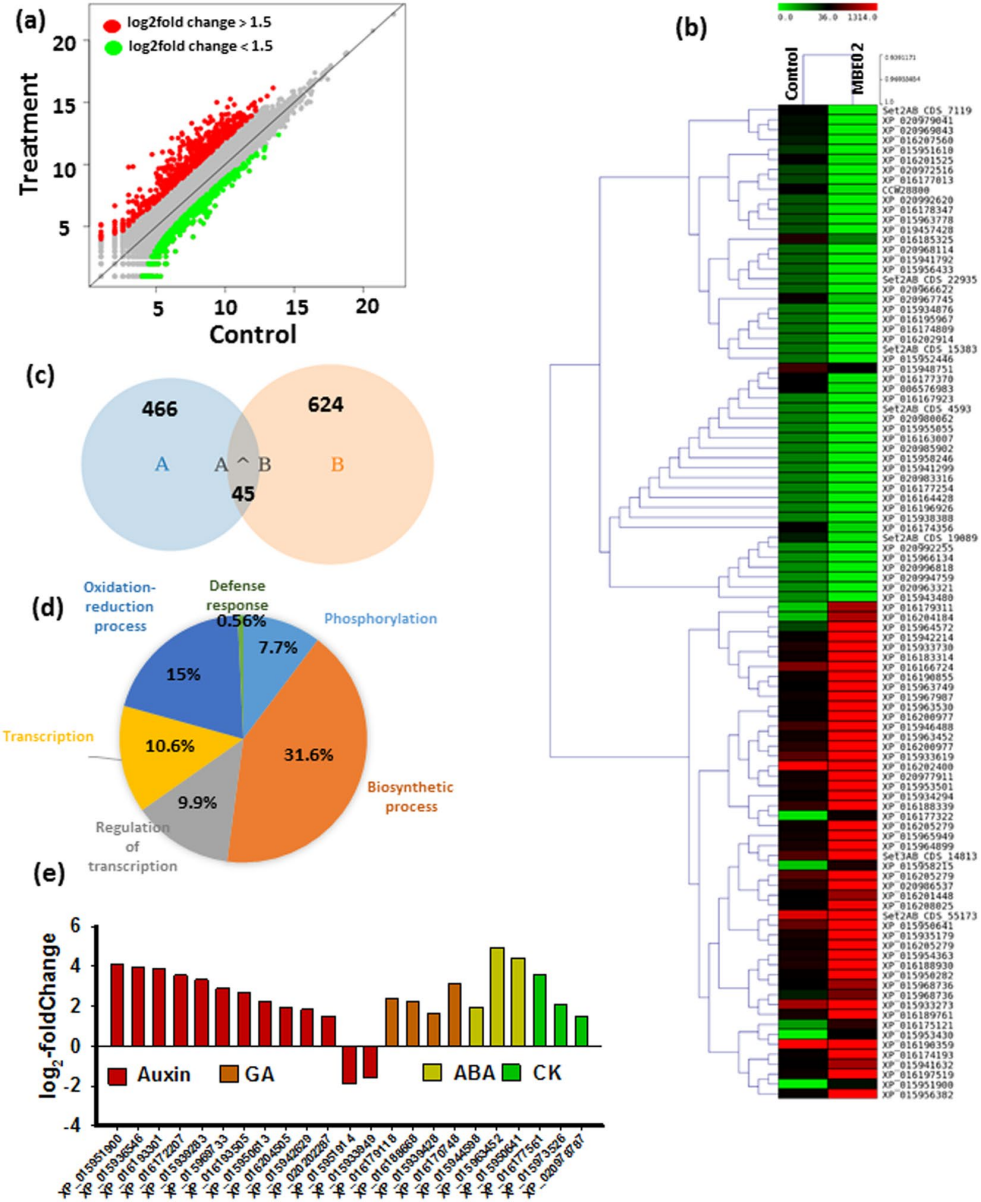
Effect of MBE02 on gene expression in peanut. (**a**) Scatter plot of differentially expressed genes between MBE02 treated roots and untreated control. 12 d old peanut plants were inoculated with MBE02 (10^6^ cells/ml) and root samples were collected after 14 days. Roots of 8-10 plants were combined for each samples. (**b**) Heat map of top 100 (50 each for up- and - down regulated) differentially expressed genes in peanut roots after MBE02 inoculation as compared to non-inoculated roots. (**c**) Venn diagram shows number of differentially expressed genes contributed by A (*A. duranensis*), B (*A. ipaensis*) and both (A + B) the progenitor genomes in MBE02 treated peanut. (**d**) Go enrichment analysis (biological category) of differentially expressed genes. (**e**) Genes involved in hormone signaling that were activated after MBE02-inculation than the control.

Differentially expressed genes (DEGs) were determined in the roots of MBE02 inoculated peanut as compared to the non-inoculated control. The abundance of each assembled transcript sequence in different samples was measured through normalized count (baseMean)^29^, and DEGs (p value < 0.05 and log2 (fold change)≥1.5≤) were defined as genes that were significantly increased or decreased in their expression levels (Fig. 2a). There were six genes that expressed in only one treatment but not the other. Of these, XP_008225167 and XP_016200422 encoded for EH domain containing protein 1 and DNA-directed RNA polymerase, respectively, whereas, others CDS were remained uncharacterized (Supplementary Fig. S3). A total of 1260 genes were differentially expressed, where 979 genes were up-regulated, whereas, 281 genes were down-regulated in bacteria inoculated seedlings as compared to control (Fig. 2a,b and Supplementary Table S3). Out of 1260, 466 genes were contributed by A genome; 624 were from B genome and, 45 genes were shared by both (Fig. 2c). The highest expression levels were obtained for calcium binding CML37 (XP_016179311) and dehydration responsive elemental binding protein 1C (XP_016204184) with 110.2 and 90.4 fold changes, respectively. Similarly, CDS 7119 (with an unidentified function) and polygalacturonase (XP_020979041), were the two most down-regulated genes as they showed a 20- and 17-fold change, respectively (Fig. 2a,b and Supplementary Table S3). We analyzed the enrichment of specific GO terms of biological process in the set of DEGs. Six GO terms comprising phosphorylation, biosynthetic process, regulation of transcription, transcription, oxidation-reduction process, and defense response were significantly enriched (Fig. 2d and Supplementary Table S4).

Hormone signaling was an important class amongst differentially expressed genes. A total of 13 auxin signaling genes were differentially expressed (Fig. 2e). Among these, six genes belong to auxin responsive SMALL AUXIN UP RNAs (SAURs) family, of which, 4 SAURs (XP_015951900, XP_015939283, XP_016193505 and XP_015950613) were up-regulated whereas, two SAUR (XP_015951914 and XP_015933949) were down-regulated compared to the control (Fig. 2e). SAURs have been implicated in regulating a wide range of physiological and developmental processes^30^. Two auxin response factors (XP_016204505 and XP_015942629), one auxin induced protein (XP_015969733) and one IAA-amino acid hydrolase ILR1 (XP_020202287) were up-regulated in MBE02 treatment. Additionally, three members of calcium binding protein PBP1 that interact with PINOID protein kinase to regulate auxin-mediated plant development^31^ were also induced by MBE02 treatment (Fig. 2e).

Further, three ABA 8′-hydroxylase genes involved in the catabolism of ABA^32^ were induced (Fig. 2e), indicating that ABA content might be decreased in MBE02 seedlings. Cytokinine synthesis (CK) genes such as cytokinin hydroxylase (XP_016177561) and cytokinin dehydrogenase (XP_015973526), were 11.8 and 4.1 fold up-regulated, respectively in MBE02 treated peanut^33^. Furthermore, gibberellin 2-beta-dioxygenase 2 (*GA2OX2*) (XP_015939428), a gene that catalyzes gibberellic acid (GA), was 3.17 fold higher than the control (Fig. 2e). The catabolism of several forms of GA is important for plant development^34^. These observations suggested that an altered hormonal balance or hormone signaling might contribute to the growth promotion of rhizobacteria-treated peanut plants that have been illustrated in Fig. 1.

**Figure 3:**
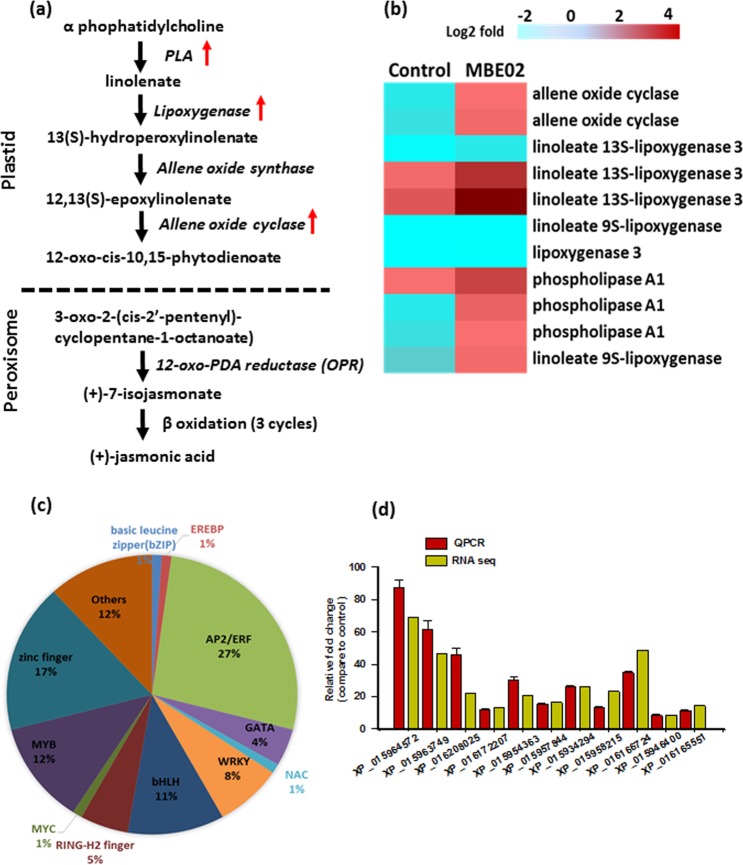
MBE02 treatment activates defense response in peanut. (**a**) Jasmonic acid (JA) biosynthesis pathway in plants, (**b**) heat map shows the differential accumulation of transcripts of JA-biosynthesis genes in MBE02 treated roots, **(c)** distribution of transcription factors in peanut roots treated with MBE02 inoculum, **(d)** quantitative real time PCR (qPCR) to validate the RNA sequencing data. Data are means ± SE (n = 3). qPCR data is in congruence with RNA sequencing data.

Moreover, MBE02 treated seedlings had increased expressions of genes related to jasmonic acid (JA) and ethylene (ET) metabolism. JA and ET are considered as stress hormones that elicit defense response in plants, and that rhizobacterial association may use these signals to prime induced systemic resistance (ISR)^5,35^. JA synthesis is initiated from linolenic acid (LA) by the action of several enzymes including lipoxygenase (LOX), allene oxide synthase (AOS), allene oxide cyclase (AOC), and 12-oxo-phytodienoic acid reductase (OPR) (Fig. 3a)^35,36^. Seven genes, (5 LOXs (XP_015939191, XP_016190077, XP_020971686, XP_015968271, XP_016205830) and 2 AOCs (XP_016181468, XP_015946400), were up-regulated; however, 1 LOX gene (XP_016170720) was down-regulated (Fig. 3b). Expression of three phospholipase A1 (PLA1) genes (XP_015963634, XP_015957844, XP_016187766) were also increased in MBE02 treated seedlings (Fig. 3b). In *Arabidopsis*, DEFECTIVE IN ANTHER DEHISCENCE 1 (DAD1), a PLA1, catalyzes the conversion of galactolipids into LA and essentially required for JA formation in pollens^37^. Besides these, 50 genes involved in biotic stress response were differentially expressed, of which 46 were up-regulated and 4 were down-regulated (Supplementary Table S5). These results indicated that the MBE02 inoculation might have also triggered ‘induced systemic resistance’ (ISR) in peanut.

A total of 93 TFs were differentially expressed in MBE02 inoculated plants. Many of them belong to ethylene responsive factor (ERF) family where 17 ERFs were up-regulated, whereas, 7 were down-regulated (Fig. 3c). ERFs are responsive to ethylene and known to play pivotal roles in regulating growth, development and stress response in various crop species^38^. Other TFs belonged to MYB (11 genes), bHLH (10 genes), WRKY (7 genes) zinc finger (16), GATA (4), 1 NAC, 1 bZIP, 1 MYC and other TF family proteins (Fig. 3c and Table S6). Among these, several TFs such as *MYB44*, *MYC4*, *ZAT10*, *ZAT11*, *WRKY30*, *WRKY11*, and *WRKY70* are reported to respond to JA^39–41^. Further, these results suggest that the rhizobacteria-mediated ISR in peanut is regulated by JA/ET signaling. The peanut *MYB72* homolog (XP_016177474, 92.5% sequence identity with *AtMYB72*), which is 2.93 fold up-regulated in MBE02 treated plants (Supplementary Fig. S4 and Table S6), is also implicated as a key regulator of ISR (Pieterse *et al*.^5^). Also, *MYC2* and β-Glucosidase 42 (BGLU42) have been implicated in ISR during beneficial bacterial association^5^. The peanut *MYC4* (XP_015963299) showed 45.7% identity to *AtMYC2* and was induced by 7.6 fold, whereas *BGLU12* (XP_016187147) had a 44% sequence identity with *Arabidopsis* BGLU42 and was induced by 3.8 fold in MBE02 treatment peanut plants as compared to the control (Supplementary Fig. S5 and Table S6). In addition, reactive oxygen species (ROS) scavenging machinery was also activated by MBE02. Our results showed that antioxidant genes such as peroxidases (5 genes up-regulated; 3 were down-regulated), glutathione-S-transferase (1 gene up-regulated), ascorbate oxidase (4 genes up-regulated) and thioredoxin (5 genes up-regulated) were differentially regulated in MBE02 inoculated plants (Supplementary Table S7).

To validate the RNA sequencing data, 17 genes were selected and their abundance patterns were analyzed with the help of quantitative real time PCR (qPCR) assays. Majority of the selected genes have functional roles in hormone signaling and/or plant defense response (Supplementary Table S8). For instance, PLA1 (XP_015957844) and AOC (XP_015946400) are components of JA pathway genes^35^; NDR1 (XP_016165551), disease resistance genes (XP_015958215 and XP_015940750), and SAR deficient 1 (XP_015954363) are involved in defense response^42^; PBP1 (XP_016172207), exordium (XP_016166724), abscisic acid 8 -hydroxylase 3 (XP_015963452) and ACC synthase like (XP_015947781) are hormone related genes^31,32,38^. Other analyzed genes are transcription factors like ERF022 (XP_015964572), bHLH18 (XP_015963749), ZAT11 (XP_016208025), DREB1C (XP_016204184) and MYB44 (XP_015934294) that controls various biological processes in the plants^43^. Most of the qPCR data corroborated with RNA sequencing data, which indicated overall quality of the Illumina RNA-Seq data (Fig. 3d and Supplementary Table S8).

Taken together, our transcriptome data demonstrated that MBE02 treatment activated genes involved in plant defenses and ISR. This raises the possibility that MBE02 treatment might have protective functions for plant to help in resisting against pathogen attack.

### Effect of *A. flavus* infection in MBE02 treated peanut

Peanut crops are susceptible for infection of aflatoxin producing *Aspergillus* species^20,44^. We determined whether activation of defense response by MBE02 treatment could help in protection of peanut from fungal infections. Peanut seedlings were inoculated with only MBE02, *A. flavus* and their combination, MBE02 + *A. flavus*, following which several growth related parameters, such as fresh weight, chlorophyll, leaf area, and plant height, were measured after two weeks of infection.

**Figure 4:**
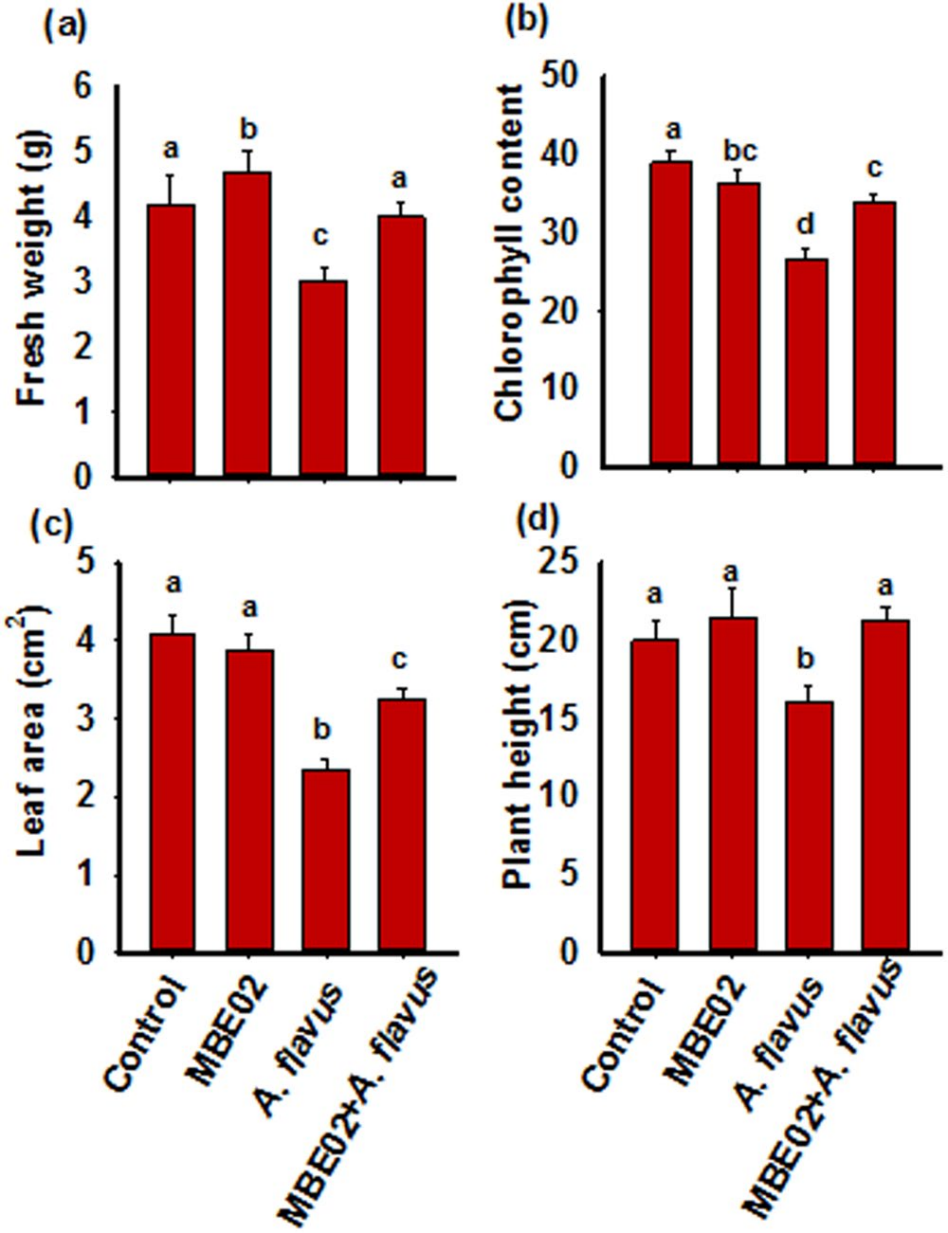
MBE02 treatment improves peanut growth in response to *A. flavus* infection. Peanut seeds were treated with MBE02 and grown up to 7 days before they were infected with *A. flavus* spore suspension (10^6^ spores/ml). Several growth-related parameters such as (**a**) fresh weight; (**b**) chlorophyll content; (**c**) leaf area; and (**d**) plant height were measured after two weeks of infection. Data are means ± SE (n = 10–12). Different letter shows statistical difference among the treatments calculated by SNK test (p < 0.05). Independent experiment was repeated that demonstrated similar results.

As expected, fresh weight was increased by MBE02 treatment^7^; however, MBE02 + *A. flavus* had no difference, and *A. flavus* treated seedlings showed 28.2% reduction than the control (SNK test p < 0.05) (Fig. 4a). Chlorophyll content was significantly reduced by 32% in *A. flavus* (SNK test p < 0.05)*;* however, it had a lesser effect on MBE02 + *A. flavus* treated seedlings which showed 13% reduction in chlorophyll content as compared to non-inoculated control (Fig. 4b). No difference in chlorophyll was observed between MBE02 and control. Similar pattern was obtained for leaf area which had 43% and 20.4% reduction in *A. flavus* and MBE02 + *A. flavus* treatment, respectively, than the control (Fig. 4c). *A. flavus* seedlings showed 20% reduction in plant height, whereas other treatments were similar as the control (Fig. 4d). Taken together our data demonstrate that MBE treatment may provide fitness benefits to plants under stressed conditions.

### MBE02 treatment increases growth and yield of peanut in presence of fungal pathogens under field condition

**Figure 5:**
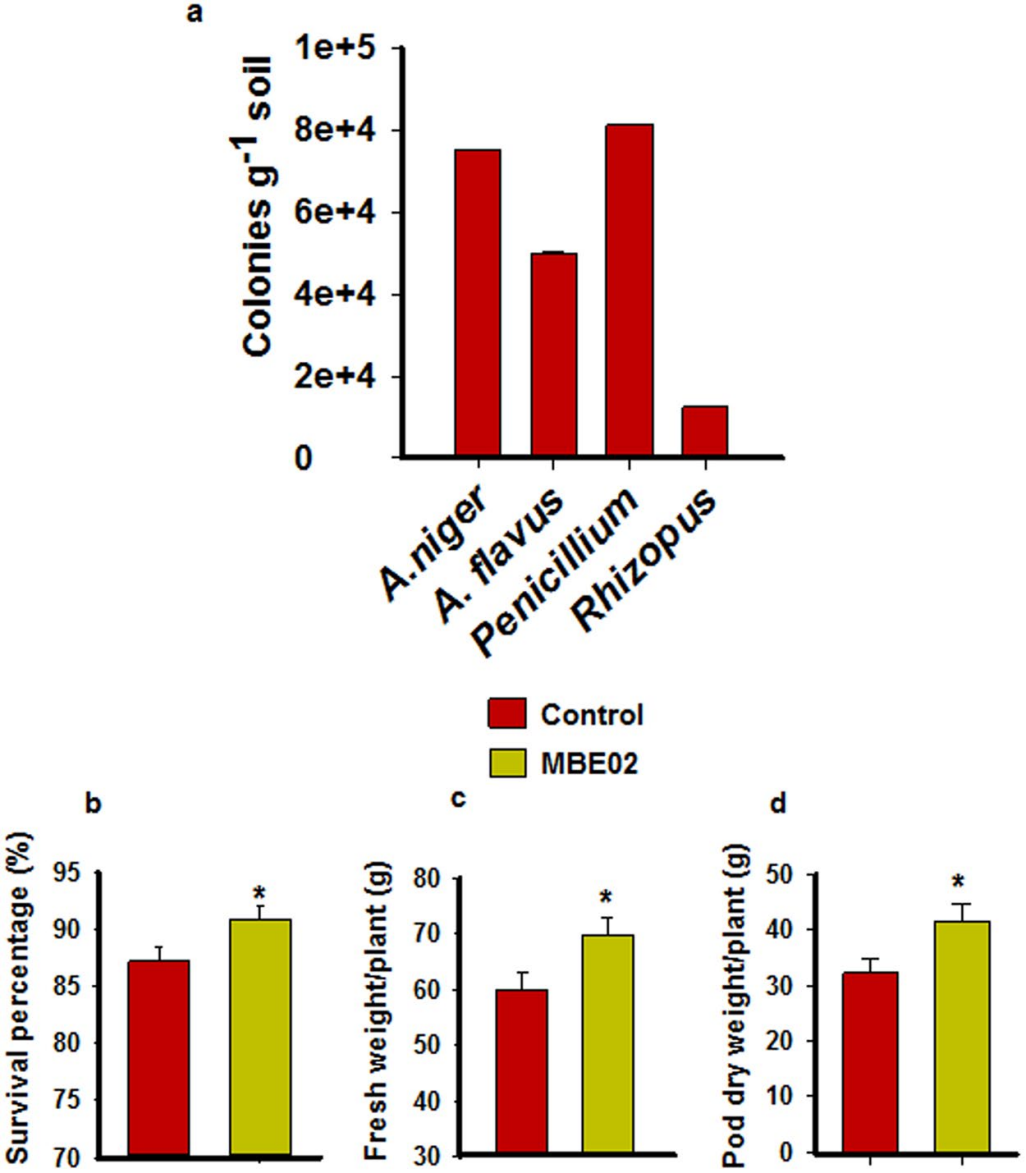
MBE02 treatment improves peanut growth and yield under field conditions. (**a**) Prevalence of fungal pathogens in the soil used for the field experiment. (**b**) Peanut seeds treated with MBE02 (O.D. 0.6) were grown in farmer’s field hired by the Banaras Hindu University, Varanasi, India and survival percentage (**b**), fresh weight (**c**) and, pod dry weight (**d**) were determined at the end of the experiment. Data are means ± SE (n = 9; where each sample contains 30-55 plants).

Our RNA sequencing data and *in vivo* infection assay indicated that the MBE02 treatment might confer resistance and provide fitness benefits to peanut plant under stressed conditions. We tested this hypothesis in natural environment by conducting a field trial where MBE02 inoculated seeds of peanut (var G20) were grown in the natural conditions in field. We determined the prevalence of fungal pathogens in the field. Soil samples were collected from different sites of the field and analyzed for the fungal frequency. The result showed presence of four fungus, *A. niger*, *A. flavus*, *Penicillium* and *Rhizopus* sp. (Fig. 5a)*; A. niger* (75000 colonies/g) and *Penicllium* (81250 colonies/g) had highest frequency in the soil, followed by *A. flavus* (50000 colonies/g) and *Rhizopus* (12500 colonies/g). Thus, as the field plot was already a hotspot for above fungi, the peanut plants were not loaded with any additional fungal inoculum. At the end of the experiment, percent survival, fresh weight and pod yield were determined.

Stress survival percentage, a ratio of live vs dead plants, was higher in MBE02 treatment than the control (Fig. 5b). Also, the MBE02 treated plants had 16% and 25% higher fresh weight (Fig. 5c) and pod dry weight (Fig. 5d), respectively, as compared to non- inoculated control (t test p < 0.05). All these observations suggested that the treatment with MBE02 confer resistance in peanut under field conditions.

### MBE02 inhibits *Aspergillus* infection in peanut seeds/pods

**Figure 6:**
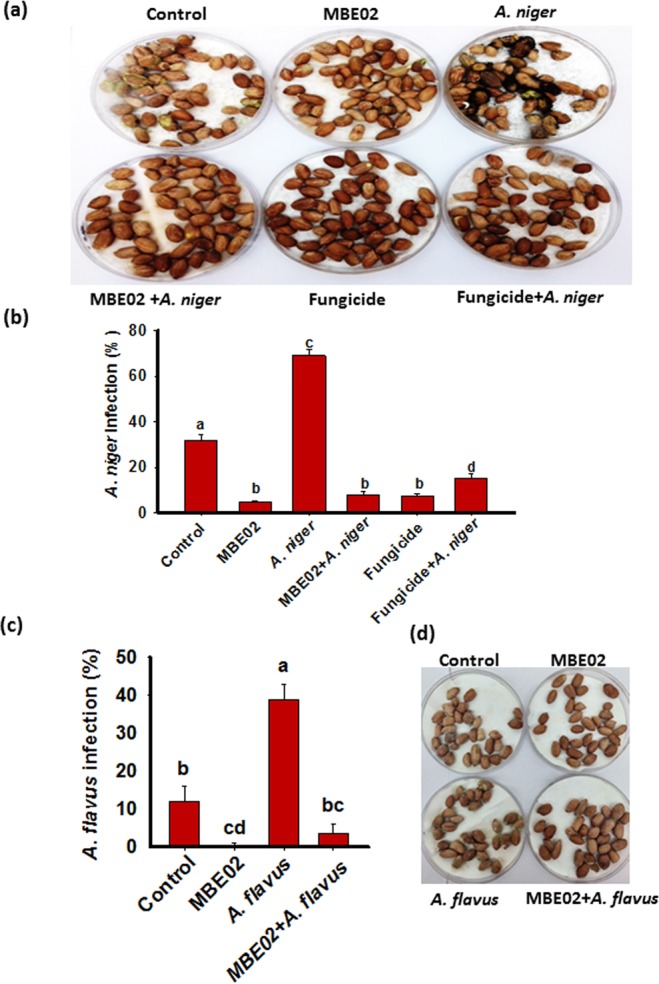
*In vitro seed* colonization assay with *Aspergillus* species in presence of MBE02. (**a**,**b**) peanut seeds were incubated with MBE02 (O.D. 0.6), *A. niger* (10^6^ spore/ml) and fungicide for 30 min. Seeds without any treatment serves as control. After incubation, half seeds from MBE02 and fungicide treatment were incubated with *A. niger* (10^6^ spore/ml) for 30 min and kept in the dark for 72 h. Data are means ± SE (n = 140-150) and combined from three independent experiments. Picture of a representative plate is shown. **(c**,**d**) Similar experimental design was used to evaluate *A. flavus* for the infection (also, here fungicide treatment was not given). Data are means ± SE (n = 40–50). Different letter shows statistical difference among the treatments calculated by SNK test (p < 0.05). Independent experiment was repeated that showed similar results.

Above studies under controlled as well as field conditions clearly demonstrated that MBE02 treatment was effective in controlling the infection of *Aspergillus* species (Figs 4 and 5). Indeed, *Aspergillus* infection is a major threat to peanut not only at pre-harvesting stage but more importantly at post-harvest, storage and during its transportation^20,21^. Hence, we investigated whether seed treatment with MBE02 prevented the *Aspergillus* infection. *In vitro* seed colonization assays were performed where fungal infections were measured on seeds that were treated either with only MBE02 or only fungus (*A. niger* or *A. flavus*) or a combination of MBE02 + *Aspergillus* pathogens. After 3 d incubation in dark, maximum infection was observed in seeds treated with only *A. niger* (70%), followed by the seeds control (no fungi, 31.8%) that might have natural loads of pathogen (Fig. 6a,b). MBE02 treatment strongly reduced the infection to 4.4% and 7.8% in MBE02 only and MBE02 + *A. niger*, respectively. The results clearly showed that treatment with MBE02 reduced the infection by 94% under standard conditions of cultivation and storage, and by 83% under conditions that might be epidemic (SNK test p < 0.05; Fig. 6a,b).

We also compared the results with a commercially available systemic fungicide that contains Carbendazim 50% WP (AIMCOZIM, India). Carbendazim is effective in preventing the infection of a wide range of fungi^45^. Seeds treated with the fungicide only reduced the infection to 7.4% whereas, the infection was reduced to 14.9% in fungicide with *A. niger* (fungicide + *A. niger*), than the seeds treated with *A. niger* only, respectively (Fig. 6a,b). Interestingly, seeds treated with MBE02 + *A. niger* had significantly less (7.8%) fungal infection than the fungicide + *A. niger* (14.9%) (SNK test p < 0.05).

We also performed similar experiments with *A. flavus*. Seed infection in *A. flavus* was highest (38.7%), followed by the controls (11.9%). The fungal infection was reduced to 0.52% and 3.6% in MBE02 and MBE02+ *A. flavus* treatment, respectively (Fig. 6c,d). The results revealed that the seeds treated with MBE02 and MBE02+ *A. flavus* had 98% and 91% reduced infection than only those infected with *A. flavus* (Fig. 6c,d), which suggested that the rhizobacteria was equally effective against other *Aspergillus* species.

We further examined if bacterial strain is effective to control *Aspergillus* infection in peanut pods. A significant reduction (SNK test p < 0.05) in *A. niger* infection was observed with MBE02 treated pods (90%) whereas, other treatments including fungicides had 43 to 49% less infection than the pods infected with *A. niger* (Supplementary Fig. S6). Overall, results obtained here demonstrated *in vitro* protection of peanut seeds/pods by MBE02 from fungal pathogens of *Aspergillus* species.

### MBE02 alters expression of lipoxygenase genes in seeds in response to *A. flavus* infection

Encouraged by the seed-pathogenesis assay, we further investigated the plausible molecular components that might be regulated by treatment of MBE02 in the seeds. Lipoxygenase (LOXs) are one of the important defense component that are known to play roles in controlling *Aspergillus* infection^46,47^. A mutant maize line of *LOX3* gene (*lox3-4*) was more susceptible for the infection of *A. flavus* and *A. nidulans* than the wild type^48^, which further supported the role of LOXs in conferring resistance against the *Aspergillus* infection in the seeds (Nayak *et al*.^21^). We performed expression profiling of *LOXs* to determine whether MBE02 reduced the *Aspergillus* infection in seeds through differential regulation of *LOX* genes. Since the peanut *LOX* encoding genes, *PnLOX2* and *PnLOX3*, are nearly identical, the qPCR assays measure the transcripts from both genes together, which are referred to as *PnLOX2-3*^46^. Additionally, 27 LOX genes have been recently identified in the genomes of two wild progenitors of peanut (*A. duranensis* and *A. ipaënsis*), few of which have been shown to respond to *A. flavus* infection^47^. Thus, we determined how the expression of these genes change in presence of MBE02 during infection of peanut seeds with *A. flavus*.

**Figure 7:**
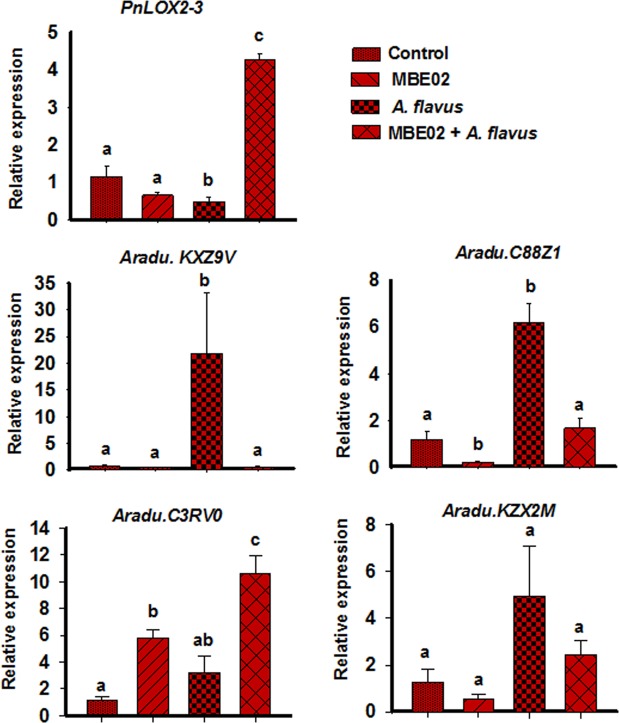
Gene expression analysis of LOX genes in MBE02 and *A. flavus* treated peanut seeds. Seeds without any treatment were taken as control (mock). Data are means ± SE (n = 3-4). Different letter shows statistical difference among the treatments calculated by SNK test (p < 0.05). Independent experiment was repeated that produced similar results.

*LOX*s displayed a complex pattern of expression after the seeds were treated with the *A. flavus* pathogen and the MBE02 bacteria. *PnLOX2-3* expression was significantly decreased in *A. flavus* infected seeds as compared to the control (SNK test p < 0.05, Fig. 7). This is consistent with the previous report^46^. Further, treatment of seeds with MBE02 alone had no effect on *PnLOX2-3* expression as compared to the only water-treated controls, whereas treatment of seeds with MBE02 and the *A. flavus* pathogen increased its expression by 8.8-fold (Fig. 7). On the other hand, expression of KXZ9V (type I 13-LOX) was induced by 21-fold in fungus infected seeds; however, other treatments (MBE02 and MBE02 + *A. flavus*) had no effect on the expression as compared to the water controls (Fig. 7). The C88Z1 gene (type II 13-LOX) was 6-fold induced in *Aspergillus* treatment; its level was significantly reduced in MBE02 only (5-fold); however, this had similar levels as in the control seeds and in MBE02 + *A. flavus* treatment (SNK test p < 0.05). The expression level of C3RV0 (9-LOX) was induced in MBE02 (5-fold) than the controls and its level was further induced by MBE02 + *A. flavus* treatment (10.6 fold). No significant induction was shown by *Aspergillus* treatment (Fig. 7). KZX2M (9-LOX) had no change in expression in any of these treatments.

Overall, above observations demonstrated that several *LOXs* were differentially regulated by MBE02 during seeds-*Aspergillus* interaction and might be involved in controlling the infection process.

### *In vitro* growth inhibition of *Aspergillus* in presence of MBE02

**Figure 8:**
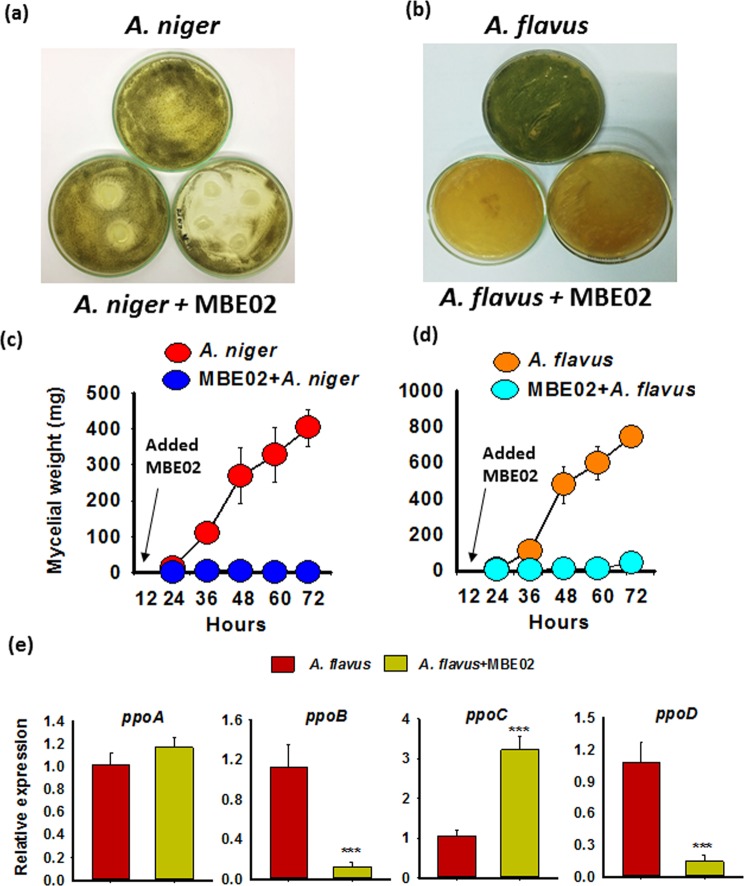
MBE02 alters growth and pathogenicity of *Aspergillus* species. Plate assays show inhibitory effect of MBE02 on *A. niger* (**a**) and *A. flavus* (**b**). Spore suspension of fungi (10^6^ spores/ml) were spreaded throughout the plate and inoculated with 5 µl of MBE02 (O.D. 0.6) followed by incubation at 25 ± 2 °C. The zone of inhibition was recorded for *A. niger* after 72 h of incubation. Representative pictures are shown here and experiment was replicated with similar results. **(c**,**d)** Time course analysis of fungal growth in presence of MBE02. *A. niger* and *A. flavus* were grown in DYGS medium and after 12 h, 1 µl of MBE02 (O.D. 0.6) was added. The fungal mycelia were harvested at the indicated time points after bacteria inoculations and weighed. Data are means ± SE (n = 3) and combined from three independent experiments. **(e)** Gene expression analysis of *Ppo* genes in *A. flavus* after treatment with MBE02. Data are means ± SE (n = 3-4). Asterisk (*) shows significant statistical difference calculated by t test (p < 0.05).

Another plausible mechanism for reduced seed infection might be that MBE02 rhizobacteria directly influenced the growth and metabolism of *Aspergillus* in peanut seeds. To test this, we performed *in vitro* growth inhibition assays where *Aspergillus* species (*A. niger* and *A. flavus*) were grown together with the MBE02 and fungal growth was monitored. MBE02 successfully inhibited the growth of *A. niger* after 72 hpi (Fig. 8a). A zone of inhibition was not appeared in case of *A. flavus*; however, MBE02 slowed down the fungal growth and delayed the sporulation as compared to control (no MBE02) (Fig. 8b). Even after seven days, *A. flavus* incubated with MBE02 had not shown any sporulation than the control (Supplementary Fig. S7). We also conducted a time course analysis where the mycelial growth of both the *Aspergillus* fungi was monitored after adding MBE02 strain. At 72 hpi of MBE02, mycelial growth of *A. niger* and *A. flavus* was reduced by 90% and 85%, respectively, as compared to the non-inoculated controls (Fig. 8c,d).

Fungal oxylipins (also known as precocious sexual inducer (psi) factor) are the secondary metabolites that originated from unsaturated fatty acids and play an essential role in the process of development and pathogenicity in *Aspergillus*^49,50^. Four psi factor-producing genes*, PpoA*, *PpoB*, *PpoC* and *PpoD*, are implicated in the biosynthesis of such fungal oxylipins, and mutant strains of these genes show defects in fungal development^49,50^. So, we determined whether growth defects in *Aspergillus* was associated with altered expression of *Ppo* genes. 12 h grown cultures of *A. flavus* were inoculated with MBE02 (1 µl of O.D. 0.6) and mycelial biomass were collected after 48 hpi for expression analysis. The time point was chosen as the expressions of all *Ppo* genes reached at their highest level at this stage^49^. *PpoA* had no change in expression, whereas *PpoB* was significantly decreased (9.1 fold) in MBE02 + *A. flavus* treatment (Fig. 8e). Similarly, *PpoD* was also reduced (−7.1 fold) in MBE02 treated fungus. On the contrary, *PpoC* expression was significantly induced by 3.21 fold in MBE02 + *A. flavus* than the *A. flavus* not treated with MBE02 (Fig. 8e). These observations indicate that MBE02 mediates dysregulation of the expression of *Ppo* genes, which might have consequences for altered growth and pathogenicity of *A. flavus*^49,50^.

### MBE02 is effective against wide range of fungal pathogens

**Figure 9:**
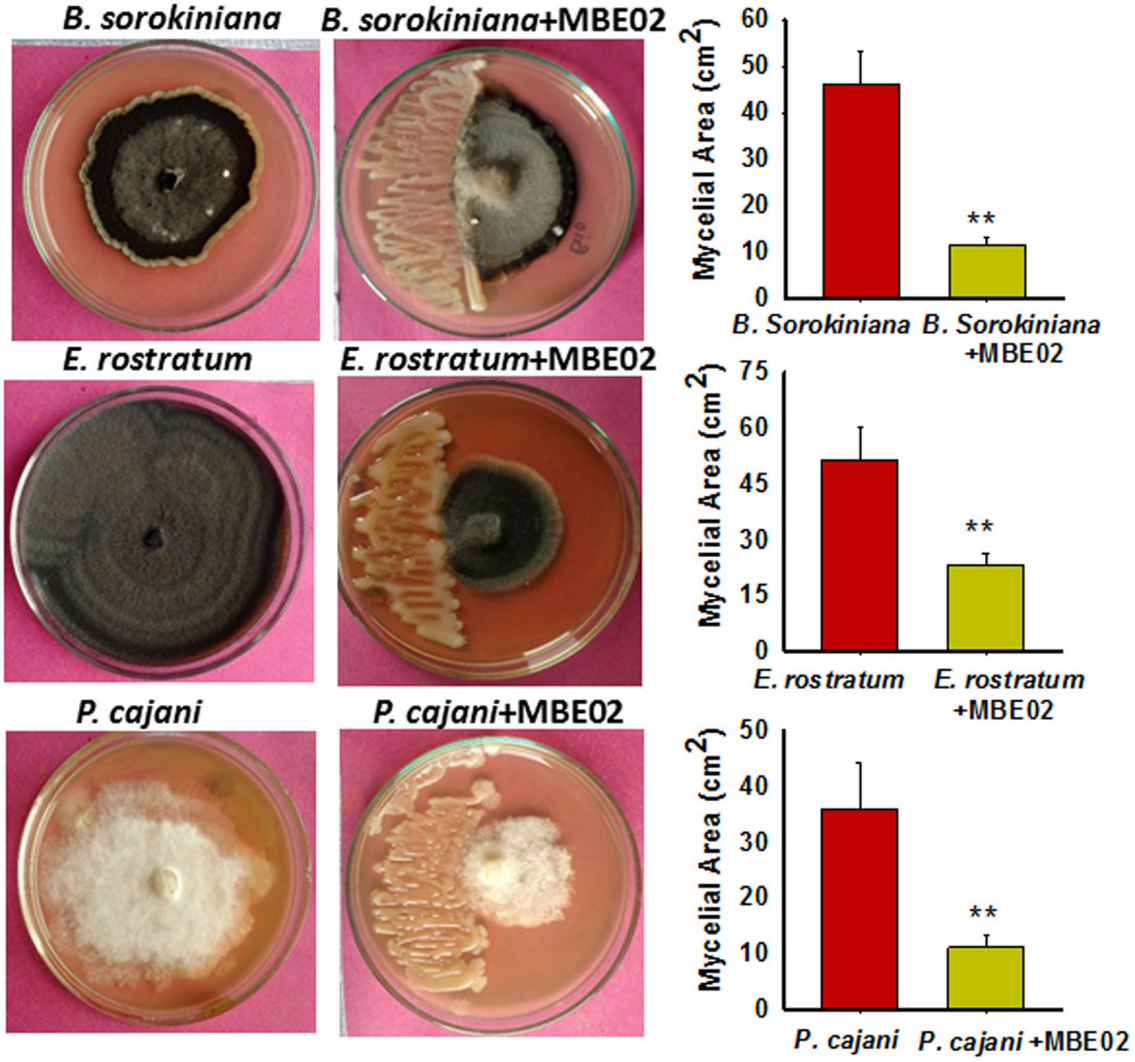
Broad spectrum effectiveness of MBE02 on growth of various fungal pathogens. Fungi were inoculated on one side of a petri dish and incubated at 25 °C. After 72 h, 5 µl of MBE02 (O.D. 0.6) was inoculated 3 cm away from the disc of the fungal colony and the zone of inhibition was recorded after 5d of incubation. Fungal plates without bacteria were taken as control. Data are means ± SE (n = 5-6) and the results were replicated in at least two independent experiments. Asterisk (*) shows statistical difference calculated by t test (p < 0.05).

We tested whether MBE02 might be effective in controlling the growth of a larger spectrum of fungal pathogens that affect different crop species. Therefore, growth inhibition assays were performed on 3 additional fungal pathogens: *Bipolaris sorokiniana* is a hemibiotrophic plant pathogen that causes spot blotch disease, and is a major threat to wheat and barley cultivation in south Asia^51,52^. *Phytopthora drechsleri* f. sp. *cajani* causes stem blight on *Cajanus cajan*^53^; *Exserohilum rostratum* is thermophillic fungal pathogens that causes leaf spot and seedling rot diseases in various plant species including wheat, rice and banana^54^. The growth of all these fungi were indeed significantly reduced in the presence of rhizobacteria (SNK test p < 0.05) (Fig. 9).

## Discussion

**Figure 10:**
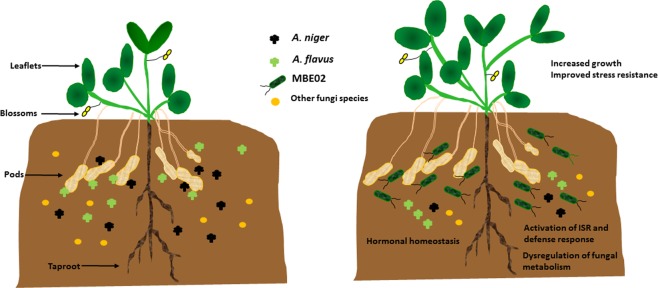
Schematic to depict the effect of MBE02 rhizobacteria on peanut under stressed conditions. (**a)** *Aspergillus* species and other fungal pathogens are present naturally in the soil where they remain in a direct contact with peanut, specially the roots and pods and cause significant reduction in its growth and yield. **(b)** Presence of MBE02 in the soil triggers host defense mechanisms that provides fitness advantage to peanut against fungal infection: (1) MBE02 alters hormonal signaling to stimulate the peanut growth and (2) trigger ISR to enhance plant immunity. (3) Additionally, being in the rhizophere, MBE02 may directly interact with pathogenic fungi trying to colonize the pods and roots, and restricts their growth by disrupting their metabolism and ability to cause pathogenicity, and in turn, confers resistance to peanut.

Root interactions with microbiota in the rhizosphere influences the health and productivity of crop plants. In this study, molecular analysis of peanut roots after inoculation with non-pathogenic rhizobacteria, MBE02, indicates that in addition to changes in growth stimulating hormonal signaling, MBE02 activates defense signaling in peanut. Our results indicate three possible ways by which MBE02 stimulates growth and protects peanut from fungi infection: (1) MBE02 alters hormonal signaling for growth promotion; (2) It triggers defense responses such as ISR that improves host resistance against fungal pathogens; (3) MBE02 can directly control the growth of seed-born pathogens as it disrupts the metabolism during its interaction with fungal pathogens. These mechanisms might independent to each other; however, a combined action of these improves peanut resistance against fungal attack and increase/or maintain the peanut growth under stress conditions (Fig. 10). Our host-pathogen interaction assays and extensive fields provide evidences for the biocontrol properties of MBE02, which can be used in preventing the growth of various fungal pathogens in several crop species.

### Rhizobacteria MBE02 activates genes associated to ISR and hormonal homeostasis in peanut

Our study demonstrated that *Klebsiella spp* strain MBE02 is a plant growth promoting rhizobacteria that can stimulate the growth of peanut under controlled/green house as well as field condition^7^ (references herein). We performed RNA-sequencing to identify the molecular components that might be effected due to association of MBE02 with plants, including deciphering those that might be involved in the growth promotion of peanut by beneficial rhizobacteria. We found that root colonization with MBE02 significantly reprograms the expression of hormonal signaling genes that might involve in growth promotion of peanut. Further, to our surprise, we further noticed that a large set of differentially expressed genes belonged to the pathways related to plant defense against pathogens, raising the possibility that MBE02 may be involved in providing immunity to plants during pathogen infection. We investigated this hypothesis.

Rhizobacteria-mediated ISR and pathogen-induced systemic acquired resistance (SAR) are two inducible defense responses in the plants that are controlled by distinct signaling pathways^5,9,10,12^. SAR is characterized by the accumulation of salicylic acid (SA) and the regulatory protein NPR1, whereas ISR requires the component of JA and ET signaling^5^. In our study, many genes influenced by JA/ET were differentially regulated, indicating that the rhizobacteria MBE02 triggers ISR in peanut through JA/ET signaling pathway. Elicitation of ISR is highly influenced by the strain type and the nature of proteins/compounds released during the host-microbe interaction. For instance, ISR induced by several strains (such as during *P. fluorescens* strain WCS417r and *Arabidopsis* interaction) is independent of salicylic acid but dependent on NPR1, JA and ET^55^. However, in other cases (such as *Bacillus amyloliquefaciens* strain MBI600 and tomato interaction), ISR is dependent on salicylic acid^56^. Induction of JA biogenesis genes in MBE02 treatment indicates that *Klebsiella* strain might alter JA homeostasis to trigger the ISR in peanut. However, this requires further investigation.

Many PGPR isolates are able to produce siderophores, hydrogen cyanide (HCN), chitinase, cellulase and other compounds. These compounds are suggested to exert biocontrol ability, restrict the growth of phytopathogens by promoting ISR, and thereby protecting the plant^3,5^. Babu *et al*. describe that PGPR pre-treated tomato plants performed better against early blight infection (caused by *Alternaria solani*), and this might be due to an enhanced accumulation of antioxidant peroxidase (POX) and polyphenol oxidase (PPO) enzymes. Such responses were further associated with the presence of siderophores, HCN, chitinase and glucanase in the isolated PGPR^57^. Similar suggestions of induced resistance in pearl millet against downy mildew disease was also made^58^. Indeed, siderophores and HCN production has been also been shown with MBE02^7^, and therefore as suggested in the studies of Jogiagh *et al*. and Babu *et al*., a plausible role of siderophore and HCN in the activation of ISR cannot be ruled out. How such compounds produced by the microbes elicit ISR and contribute to plant defense in peanuts requires further investigation.

Although ISR activates defense signaling components to protect the plants from the attack of various pathogens^57,58^, it does not produce negative impact on the plant fitness. This means beneficial rhizobacteria have evolved such a mechanism that reduces the cost of defense signaling^5,6^. One of such mechanism is the activation of auxin signature in the host plants^2,3,57,59^. Activation of auxin signaling is essentially required not only to stimulate growth but also to activate ISR in *Arabidopsis* roots^6^. In our study, several auxin responsive genes were found activated in MBE02 plants. We hypothesize that this beneficial rhizobacteria might use auxin signaling to maintain a balance between growth promotion and elicitation of systemic immunity in peanut. Additionally, other hormone genes were also differentially regulated by MBE02, which suggests that MBE02 regulates these genes to alter the hormonal homeostasis that may lead to improved peanut performance.

### MBE02-elicited transcriptional regulators of ISR in peanut roots

Apart from the contribution from JA/ET signaling, other regulators that control ISR were also activated in peanut. *MYB72* is an important component of early signaling in rhizobacteria mediated ISR, whereas *MYC2* and the *BGLU42* β‐Glucosidase works downstream to *MYB72* during the interaction between *Arabidopsis* and WCS417 strain^15–17^. In addition, *WRKY11* and *WRKY70* were identified as important regulators of ISR triggered by *Bacillus cereus AR156*^60^. In the peanut transcriptome, we identified 11 MYB transcription factors, of which, *MYB108* had 92.5% sequence identity with *AtMYB72*, which indicates that *MYB108* might have similar functions in activating ISR in peanut. Similarly, *MYC4* and *BGLU12* β‐Glucosidase in peanut showed close similarity with their orthologue partners (*MYC2* and *BGLU42*) in *Arabidopsis*. It is noteworthy that *MYC4* and *BGLU12* are the only members of their family that were specifically differentially expressed by MBE02 treatment, indicating they might have important roles to play in MBE02-induced ISR in peanut. In addition, peanut also expressed *WRKY11* and *WRKY70* in MBE02 treatment indicating that these signaling components play similar roles in different plant species in response to beneficiary microorganisms.

Recently, Hao *et al*.^61^ performed a transcriptome analysis of peanut roots treated with beneficial and pathogenic fungi. In contrast to our observation, their study has found relatively fewer number of DEGs^61^. This could be due to a difference in the experimental design between the two independent studies, including the use of different peanut genotypes, microbes and time points for the sample collection. Despite these differences, both studies shared common genes (few examples are *MYC4*, *ZAT11*, *DREB1C*, *WRKY41*) that are differentially regulated in peanut during inoculation with beneficiary microorganisms of different groups. These observations indicate that mentioned molecular components might have key roles in interaction between peanut and beneficiary microorganisms.

### Role of LOXs in controlling *Aspergillus* infection in MBE02 treated peanut seeds

*In vitro* seed colonization assays revealed that MBE02 efficiently prevented the growth of *Aspergillus* pathogens. Transcript analysis of infected seeds showed differential expression of various LOX genes in response to MBE02 inoculation. LOX genes produce different ratios of *cis-trans* 9S- or 13S-hydroperoxy linoleic acid (9S- or 13S-HPODE)^47^, that are metabolized by different pathways to yield various oxylipin products, some of which activate or repress *Aspergillus* differentiation processes, including spore development, sclerotial development, and mycotoxin biosynthesis^46,49,50,62^. Several lines of evidences suggests that 9-LOX-derived oxylipins increase aflatoxin production, whereas 13-LOX-derived oxylipins inhibit mycotoxin synthesis^63^. Furthermore, a decreased content of 13-HSPDE in *Aspergillus* infected seeds was well correlated with repressed expression of *pnlox2-3*^46^. It is plausible that increased expression of *pnlox2-3* in MBE02 + *A. flavus* infected seeds will contribute to the overall content of 13-HPODE content and led to decreased *A. flavus* infection and aflatoxin production. Moreover, differential regulation of other LOX genes in the infected seeds might contribute to the resistance against *Aspergillus* infection. The function of these genes in controlling *Aspergillus* infection in peanut seeds require further investigation.

### MBE02 de-regulates development/pathogenicity factors in *Aspergillus flavus*

MBE02 might disrupt components essential for the processes of development and aflatoxin production in *Aspergillus*, such as the *Ppo* genes. Expression of *Ppo* genes were altered in *A. flavus* in presence of MBE02. The conserved *Ppo* oxygenase genes produce oxylipins in fungi that are physiologically and structurally similar to plant oxylipins^64–66^. It has been proven that the oxylipins of these *Ppo* genes are essentially required for the transition from one developmental stage to another in *Aspergillus*. Single or multiple mutants of *Ppo* genes showed developmental defects and inability to colonize the peanut seeds^46,64,66,67^. It is postulated that MBE02 inhibits the development and virulence of *Aspergillus* by dysregulating the expression of *Ppo* genes and, therefore, reduce the *Aspergillus* infection in peanut seeds.

Overall, our study provides molecular insights into MBE02-triggered response of peanut and show that MBE02 can work as an effective biocontrol agent against a wide range of fungal pathogens of peanut as well several other crop species. Future research would warrant, (1) functional validation of candidate genes identified from RNA sequencing of bacteria inoculated peanut roots, and (2) release of MBE02 as a biocontrol agent for better disease management of peanut and other important crops.

## Methods

### Sample collection and preparation of *Arachis hypogaea* L. roots

Plant material and growth conditions were same as described previously^7^. As MBE02 demonstrated growth promoting properties^7^, this strain was investigated further for investigation. For RNA sequencing, 12 d old peanut seedlings were treated with MBE02 (10^6^ cells/ml) and root samples of 8-10 different plants/samples were collected after 14 days of treatment, immediately frozen into liquid nitrogen, and stored in −80 °C until further use. Seedlings not inoculated with bacteria were taken as controls. Two biological samples were collected for each treatment.

### RNA extraction, library construction and sequencing

Total RNA was isolated by using the RNAeasy plant mini kit with column DNase digestion (Qiagen, Hilden, Germany) following the manufacturer’s instructions. RNA concentration was then measured using Qubit RNA Assay Kit in Qubit 2.0 Flurometer (Life Technologies, Carlsbad, CA, USA). Also, RNA integrity was assessed using the RNA Nano 6000 Assay Kit of the Bioanalyzer 2100 system (Agilent Technologies, Santa Clara, CA, USA). Samples with RNA integrity number (RIN) values above 8 were used for construction of the libraries.

Sequencing libraries were generated using NEXTflex™ Rapid Directional RNA‐Sequencing Kit for Illumina (BIOO Scientific, USA) following manufacturer’s recommendations and index codes were added to attribute sequences to each sample. 2 μg of total RNA was taken for Poly-A enrichment. The enriched mRNA was fragmented, primed and reverse transcribed using First Strand Synthesis Mix. Second strand DNA was synthesized and ends were repaired using Second Strand Synthesis Mix. Double stranded cDNA was purified using High Prep PCR magnetic beads (MagbioGenomics Inc, USA), adenylated and ligated to Illumina multiplex barcode adapters. The adapter ligated cDNA fragments were purified, and subjected to PCR for enrichment. PCR product (sequencing library) was purified followed by library quality control check. The Illumina‐compatible sequencing library was initially quantified by Qubit fluorometer (Thermo Fisher, USA) and fragment size distribution was analyzed on Tape Station (Agilent Technologies, Germany). The libraries were pooled in equimolar amounts to create final multiplexed library pool for sequencing on an Illumina Next Seq500 with 75 bp paired‐end chemistry.

### Assembly, mapping and functional annotation of sequenced reads

Clean reads were obtained by eliminating the adapter sequences, ambiguous reads (reads with unknown nucleotides “N” larger than 5%), and low-quality sequences (reads with more than 10% quality threshold (QV) < 20 phred score) than 10% quality threshold (QV) < 20 phred score) were eliminated. The quality of raw and filtered reads was assessed via FastQC. To generate peanut reference transcriptome sequence, reads were aligned to the genome of peanut progenitors, *A. duranensis* (A genome) and *A. ipaensis* (B genome)^18^, using Tophat^68^ and, aligned sequences were assembled with trinity using genome guided strategy as described^69,70^. Gene function was annotated by using NCBI non-redundant protein database (Nr) database using Basic local alignment search tool (Blastx) (E-value: *1e-05*).

### GO and KEGG Pathway analysis

Gene ontology (GO) annotations were determined by the Blast2GO program. GO assignments were used to classify the functions of the predicted CDS. To identify the potential involvement of the predicted CDS of the control and treated samples in biological pathways, CDS were mapped to reference canonical pathways in KEGG. The KEGG Orthology database of *Arachis* genus was used as the reference for pathway mapping. The output of KEGG automated annotation server KAAS (http://www.genome.jp/kaas-bin/kaas main)^71^ includes KEGG Orthology (KO) assignments with corresponding enzyme commission (EC) numbers and metabolic pathways of predicted CDS.

### Differential expression analysis,

Differential expression analysis was performed between control (Set2AB) and MBE02-treated (Set3AB) samples by employing a negative binomial distribution model in DESeq package^72^ (version1.22.1-http://www.huber.embl.de/users/anders/DESeq/). The package was used to normalize count data and calculate mean values (baseMean, baseMean control and baseMean treatment) as described^29^. Dispersion values were estimated with the following parameters: method = blind, sharing Mode = fit-only and fit type = local. Log_2_-fold change (FC) value was calculated with the help of SAM tools, by computing read counts using the formula, $Fc=Lo{g}_{2}(treated/control)$. P-value threshold of 0.05 was used to filter statistically significant results.

### Effect of MBE02 against *A. flavus* infection in peanut seedlings

*In vivo* pathogenesis experiment was conducted in controlled conditions of a greenhouse (16 h light/8 h dark; 26–28 °C, light intensity of 100–150 µmol photons m^−2^ s^−1^). Peanut seeds were incubated with rhizobacteria (0.6 O.D. ~10^8^ cells/ml) for 1 h before transfer to the soil pots. After a week, seedlings were infected with 20 ml spore suspension of *A. flavus* (10^6^ spores/ml) and various growth related parameters (chlorophyll content, plant height, leaf area and fresh weight) were analysed after two weeks of infection.

### Soil analysis and plant pathogenesis assay under field condition

The experiment was conducted during July to November 2017 at the farmer’s field hired by the Banaras Hindu University, Varanasi, India (25.1048°N, 82.9247°E). First, the fungal frequency was determined in the soil. Different samples were collected from the fields and processed by soil plated method^73^. Briefly, soil was transferred into a sterilized petri dish and 8-10 ml of cooled potato dextrose agar medium (PDA) medium was added and incubated at 25 °C. After 6 days of inoculation, number of fungal and bacterial colonies were counted and identified based on their morphological characters.

Peanut seeds (var. G20) were treated with MBE02 suspension (O.D. 0.6) for 1 h before they were placed in the soil. Row-to-row and plant-to-plant distances were kept at 45 × 30 cm. Whole experiment was conducted in a randomized block design with plot size 5 × 3 m that was replicated three times. Plants were not treated with any artificial infection of the pathogens. Seeds not inoculated with bacteria were taken as controls. Stress survival percentage, plant weight and pod yield were determined at the end of the experiment. Standard agronomic practices were followed throughout the experiment.

### *In vitro* seed colonization assay

Peanut seeds (var. G20) were incubated with different treatments (control, MBE02 (O.D. 0.6), fungus (*A. niger*, 10^6^ spores/ml) and, fungicide (carbendazim 50% WP)) and kept on a shaking incubator for 30 min. After incubation, half of MBE02 and fungicide treated seeds were mixed with fungus for MBE02 + *A. niger* and fungicide + *A. niger* treatments, respectively, and, incubated for another 30 min on a shaking incubator. Similar experiment was repeated for *A. flavus* treatment. It is noteworthy that control seeds were not surface sterilized as one of our objectives was to test whether MBE02 could prevent all kinds of infections that usually comes from the pathogens naturally present in the seeds. After treatment, seeds were wiped with the sterile tissue and transferred into Petri plates already moistened with layer of three whatman filter papers. Seeds were analyzed for the fungal infection after 3 days. Fungal infection was scored between 0-4 scales where 0 scored no (0%) and 4 scored 100% infection. Three to four independent experiments were performed.

### Growth inhibition assay of fungal pathogens in presence of MBE02

A total of five fungi *viz., Aspergillus niger, A. flavus, Bipolaris sorokiniana (strain HD3069), Exserohilum rostratum*, and *Phytophthora dreschleri f. sp. cajani* were subjected to the antagonistic test in growth inhibition assays. A disc of 2 mm diameter of fungus was placed on one side of a Petri dish and incubated at 25 °C. After 72 h of fungal growth, bacterial strain (MBE02, 5 µl of O.D. 0.6) was inoculated 3 cm away from the disc of the fungal colony. For the fast-growing fungi like *A. niger or A. flavus*, inoculation was done simultaneously with the bacterial streaking on the same plate followed by incubation at 25 ± 2 °C for 72 h. Plates inoculated with only pathogens served as controls. The experiment was repeated twice with 5-6 replications for each treatment. The zone of inhibition was recorded by measuring colony area after 72 h incubation for *Aspergillus spp*, and after 5 d of incubation for rest of other fungi, by Adobe Photoshop Version: 12.0. Percent inhibition was calculated using the following formula:

$$ \% \,{\rm{inhibition}}=[{\rm{1}}-(\mathrm{Treatment\; growth}/\mathrm{Control\; growth})]\times {\rm{100}}$$

For inoculations in liquid culture, 10 ml DYGS medium was inoculated with the fungi (*A. niger* and *A. flavus*) and grown at 28 °C in a shaking incubator at 180 rpm. After 12 h, 1 µl bacterial inoculum (O.D. 0.6) was added, and fungal growth was measured at different hours of post inoculation (hpi). Fungal cultures without adding rhizobacteria were taken as a controls. Three tubes were grown for each time point and experiment was repeated at least three times.

### Quantitative Real Time PCR

Peanuts treated with MBE02 bacteria and *A. flavus* were collected for RNA isolation. Total RNA was isolated with Plant RNAse kit (Qiagen) with on-column DNAse treatment and quantified with NanoDrop™ spectrophotometer (Thermo scientific, USA). 0.5-1 µg of total RNA was reverse transcribed by using ImProm-II™ reverse transcription system (Promega, USA). For real time analysis, 3 fold diluted cDNA was used in a reaction mixture with PowerUp SYBR™ Green Master Mix (Invitrogen, USA) and run in a Real-Time iQ5 Cycler (Bio-Rad, USA)^7^. Three biological samples for each treatment were processed, and the reaction was set up in duplicates^74^. Actin gene was used as an endogenous control to normalize the gene expression. Relative fold change was determined by 2^−ΔΔCt^ method^75^.

For the experiment of MBE02 and *A. flavus* interactions, fungal mycelia were collected for RNA isolation after 48 h of bacteria inoculation. RNA isolation and real time PCR was conducted as described above. Three biological samples for each treatment were processed. Gene expression values were normalized with actin gene. The details of the primers used in the study are given in the Supporting Information Table S9.

### Statistical analysis

Data analysis was performed with the help of IBM SPSS statistics 19. Treatment means were compared by Student-Newman-Keuls (SNK) test at 5% probability (p < 0.05). For *in vitro* seed colonization experiment, values were converted into percent fungal infection after they were compared by SNK test. In some cases, significant differences were calculated by t test at 5% probability (p < 0.05). Data are represented as means ± SE (standard error).

## Supplementary information

Supplementary information

Table S1

Table S2

Table S3

Table S4

Table S5

Table S6

Table S7

Table S8

Table S9

## Data Availability

RNA sequencing data has been deposited at NCBI with accession number GSE125807.
