# Supplementary material for: A halotolerant growth promoting rhizobacteria triggers induced systemic resistance in plants and defends against fungal infection
Source: Sci Rep. 2019 Mar 11;9:4054. doi: 10.1038/s41598-019-40930-x (PMC6411892; doi:10.1038/s41598-019-40930-x)
Supplement: Supplementary file 1 — Supplementary information [file 41598_2019_40930_MOESM1_ESM.pdf]

Supplementary information

Title:

A halotolerant growth promoting rhizobacteria triggers induced systemic resistance in plants and defends against fungal infection

Sandeep Sharma[1ǂ], Chen Chen[2], Sudhir Navathe[3], Ramesh Chand[3], Shree P. Pandey[4]

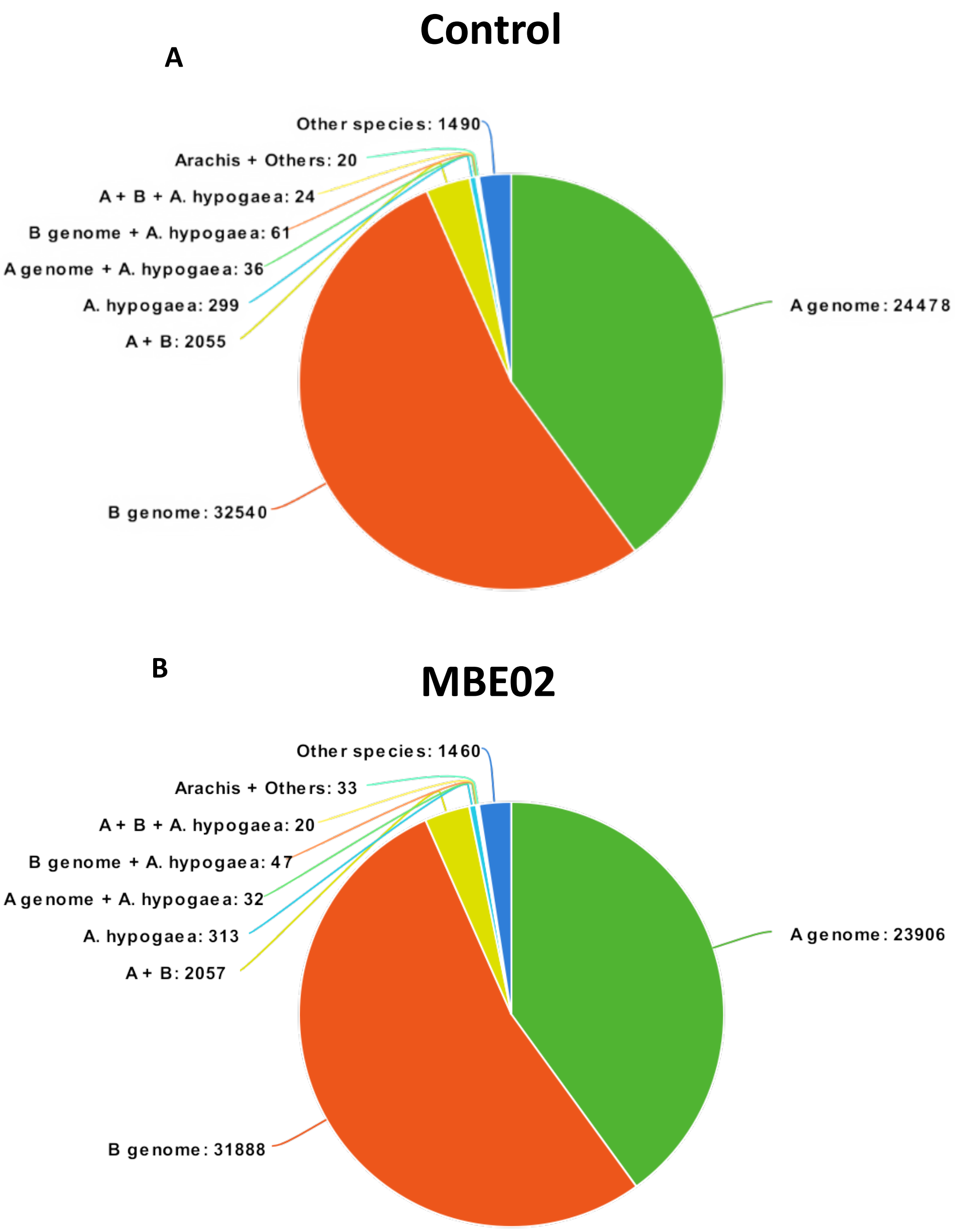

Figure S1: Pie chart shows blast hit distribution of CDS of control **(a)** and MBE02 samples **(b). '**A' and 'B' genomes represent *A. duranensis* and *A. ipaensis*, respectively.

A

Control

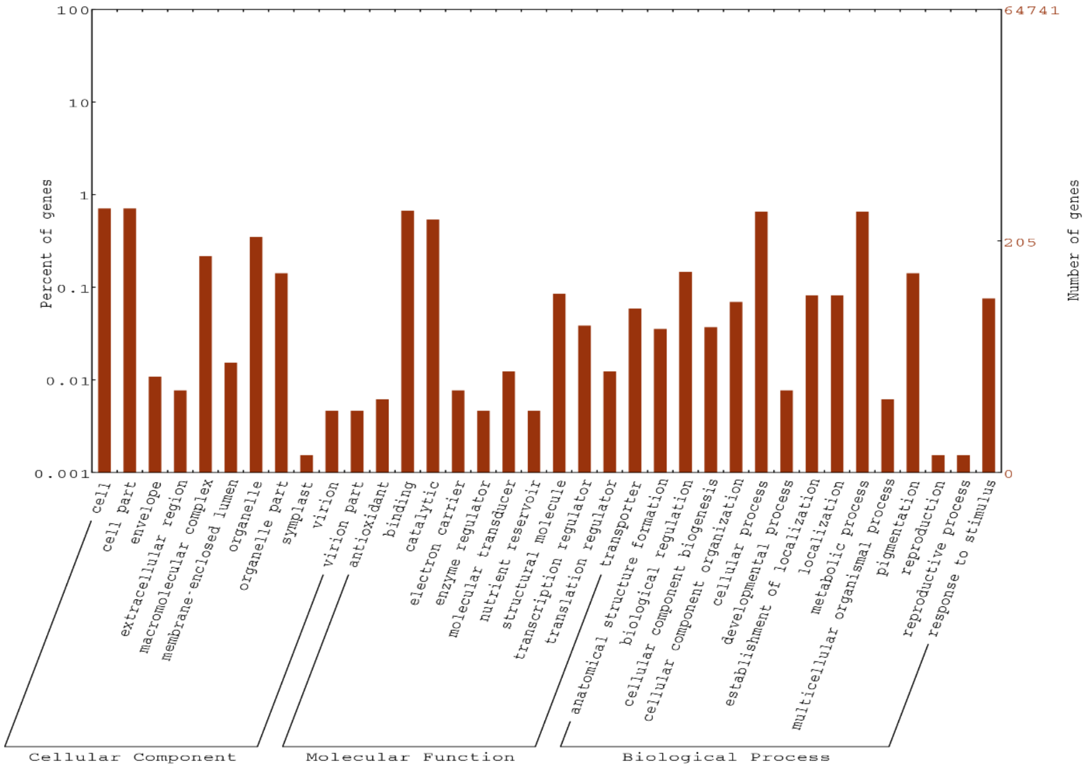

B

MBE02

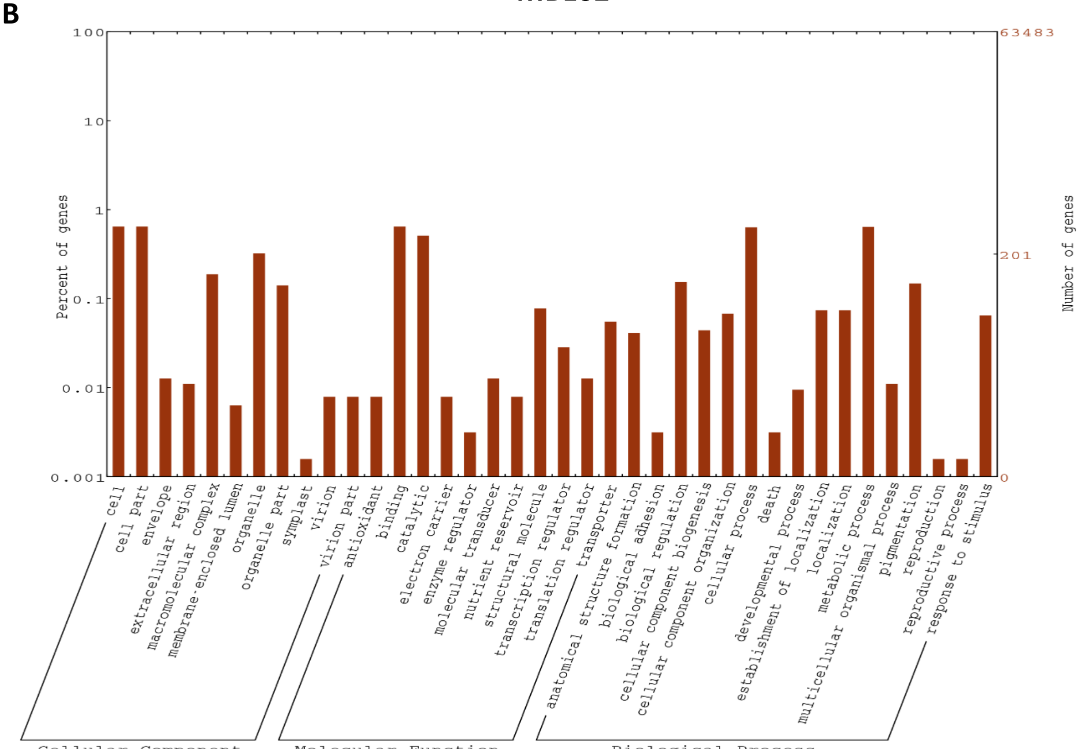

Figure S2: GO enrichment analysis of control and MBE02-treated samples. WEGO plot shows distribution of GO terms across three categories (biological, cellular and molecular functions) in control (**a**) and MBE02 treatment (**b)**

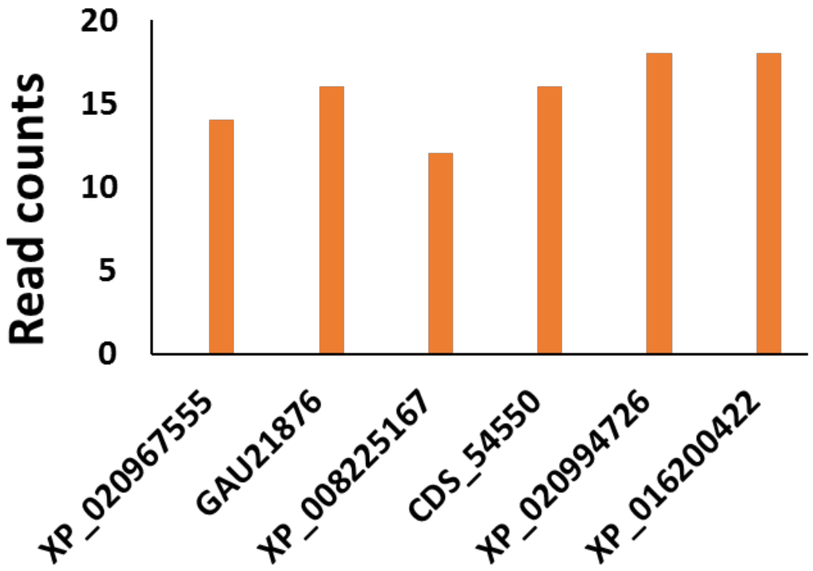

Figure S3: Read counts of CDS that expressed in MBE02-treatment but not in the control. XP_008225167 encodes for EH domain containing protein 1 and XP_016200422 encodes for DNA-directed RNA polymerase. Other four CDS remain uncharacterized.

```
XP_015953561.1      ---------------------MVRAPFYDKYGVKKGAWSREEDQRLIAFVQRYGHSNWRQ
AtMYB72             -------------------MGKGRAPCCDKNKVKRGPWSPQEDLTLITFIQKHGHQNWRS
XP_020984775.1      ---------------MMSKDKEEEKKNKSKNKLRKGLWSPEEDEKLMRYMMNNGQGCWGE
XP_016177474.1      MEMEYTSSVHMIMRSLSDCGSSVTNGSEEDMEIRKGPWTEEEDSALLNHITTYGEGHWNS
XP_016201689.1      ----------------MNTATSTTAVTRKDMDRIKGPWSPEEDDALQKLVEKHGPRNWSL
XP_015972562.1      ------------------------------MDRVKGPWSPEEDETLRRLVGAHGPRNWTI
XP_015934294.1      MEALNNRSSSCTASSSDSSSSESTQRNPNRPERIKGPWSAEEDRILTRLVERYGARNWSL
                                                      :* *: :**  *    :    *    *

XP_016188339.1      LPKFAGLARCGKSCRLRWMNYLRPNLKRGNFTQEEEACIIKLHSKLGNRWSAIAAELPGR
XP_015953501.1      LPKFAGLARCGKSCRLRWMNYLRPNLKRGNFTQEEEACIIKLHSKLGNRWSAIAAELPGR
XP_015953561.1      LPKFAGLARCGKSCRLRWLNYLRPNLKHGNYTQEEEELIIKLHQQLGNRWSLIAERLPGR
AtMYB72             LPKLAGLLRCGKSCRLRWINYLRPDVKRGNFSKKEEDAIIHYHQTLGNKWSKIASFLPGR
XP_020984775.1      VARNAGLQRCGKSCRLRWINYLRPDLKRGAFSPQEQQLIIHLHSLLGNRWSQIAAQLPGR
XP_016177474.1      VARSAGLKRSGKSCRLRWLNYLRPNVRRGNITLEEQLLILDLHSRWGNRWSKIAEQLPGR
XP_016201689.1      ISKSIPG-RSGKSCRLRWCNQLSPQVEHRAFTPEEDDTIIRAHARFGNKWATIARLLSGR
XP_015972562.1      ISKSIPG-RSGKSCRLRWCNQLSPEVEHRAFTPEEDDIIVRAHAEFGNKWATIARLLNGR
XP_015934294.1      ISRYIKG-RSGKSCRLRWCNQLSPTVEHRPFSSHEDDTIIAAHAQYGNRWATIARLLPGR
                    :.:     *.******** * * * :.:    : .*:  *:  *    **:*: **  * **

XP_016188339.1      TDNEIKNHWHTTLKKRFQTNEEESETATKSKPKETTQISAESNTTTTSPLSPLSSSSEFS
XP_015953501.1      TDNEIKNHWHTTLKKRFQTNEEES------KPKETTQISAESNTTTTSPLSPLSSSSEFS
XP_015953561.1      TDNEIKNHWHSHLKKLSSKNNNNEINPSSESESESKSNNPIDDDILEGKHTNQPKDSVSP
AtMYB72             TDNEIKNVWNTHLKKRLTPSSSSSSLSSTHDQSTKADHDKNCDGAQEEIHSGLN-ESQNS
XP_020984775.1      TDNEIKNFWNSTIKKRCITITSSSTSQNTSESYSSWLEPHDHHNNMEGFTMMPMFSCSSS
XP_016177474.1      TDNEIKNYWRTRVVKQAKQLKCDVNSKQFRDALRYVWMPRLIEQIQAQGRSTMCVSQARE
XP_016201689.1      TDNAIKNHWNSTLKRKCASMGPIDDPHFAQPLKRSVSAG--AAVPVSTGLYMNPPTPGSP
XP_015972562.1      TDNAVKNHWNSTLKRKCRSTASDAVTVAVYPDAQPLKRS--ASVGP-----CHVTTPSSP
XP_015934294.1      TDNAVKNHWNSTLKRRARDQRRGGSATGVHATCASLAAAPTNER-------------ASC
                    *** :** *.: : :

XP_016188339.1      SITSWDQSHNNNN--KNNMV------LEDDDFAFLDGSFWTEPYLAEILYE---------
XP_015953501.1      SRTSWDQSHNNNNNNKNNLV------LEDDDFAFLDGSFWTEPYLAEILHEQETVVTVPS
XP_015953561.1      SYDYVDSANNNKFQHRVLES------SSSTETSSYTDDEVGHSYYSLTPNNVEQSTIMNN
AtMYB72             ATSSHHQGECMHTKPELHEV------NGLNEIQFLLDHDDFDDITSEFLQDNDILFPLDS
XP_020984775.1      QSPSIIIDEHLPMPMENNNM------HAAMGINNNNNNMFVPPLLESATTSNVYFDEDVN
XP_016177474.1      IVNPVTSVSMASSKSCSSFS------GCEQFQASSSVSDSCVSYYSLMGSGSGGGSSE--
XP_016201689.1      SGSDVSESSVPAASTSHVFRPVPRAGGVVPPVETTSSSYDPPTSLSLSLPGVDSNSEVSN
XP_015972562.1      SGSELSDPGLPSLST---------------------PGSDPRTLLSLSLPGSSGSGAGSD
XP_015934294.1      SSGALPPCTLP-------------------------LEDDPLTALTLAPPGIDRGS----

XP_016188339.1      ------------------------------------------------------------
XP_015953501.1      STSNVFDPCEISVANENEELELSSNNSTSSLFDLEKEFGSFLIEPTMMESFWTQPCVSES
XP_015953561.1      SPTSSEDYCVVPSVSFEEFGG----------------------------DFWTEPFIVAN
AtMYB72             LLHNHQTHISTQEMTREVTKSQSFDHPQPD-----------------IPCGFEDTNEESD
XP_020984775.1      TLHN--------------------------------------------KVVSVEEDSLIS
XP_016177474.1      ---------------------------------------------------HAEKGATSS
XP_016201689.1      RGNEPSPHSAPPLTAIPLLPIMSAAVSATVAAVPAVTATAPGTAPLAAQPLQQIRDPSAV
XP_015972562.1      ----------------------------------------------------SIKDGLGS
XP_015934294.1      -----------------------------------------------------------G

XP_016188339.1      ------------------------------------------------------------
XP_015953501.1      DDVSHIPFMPPSSEYFTTLCGSDPWSTPSTTHLYDQHLSLFH------------------
XP_015953561.1      NDAYANDEIIISGEDDVGFLVPYLYESADFCFNL--------------------------
AtMYB72             LRRQLVESTTPNNEYDEWFNFIDNQTYFDDFNFVGEVCL---------------------
XP_020984775.1      MEDWELEDLMIKSVSSPFLDFPLS------------------------------------
XP_016177474.1      STPFEPENGFGGADLWTDENIWFLQQQLADDDHL--------------------------
XP_016201689.1      AAVPKQNNGAAPFNFSAELLAVMQDMIRKEVRSYMAGLEQQSNNNGMCFQAADGGFRNAS
XP_015972562.1      GLCPDPD---PAQMFSPEFLAMMQEMVRKEVRNYMSMVEENG-----VRMQQTEAIRNGV
XP_015934294.1      AMEAEQQRASPETSVGSGFWDVMRDVIAREVREYVSSN-----------FSDHSNFH---

XP_016188339.1      ------------
XP_015953501.1      ------------
XP_015953561.1      ------------
AtMYB72             ------------
XP_020984775.1      ------------
XP_016177474.1      ------------
XP_016201689.1      VKRIGISRIDS-
XP_015972562.1      MKRMGIRNGLER
XP_015934294.1      ------------
```

Figure S4. Pair-wise sequence alignment of MYB sequences (up-regulated in MBE02 treatment) of peanut with *Arabidopsis* MYB72 (*AtMYB72*). MYB108 like (XP_016177474.1) shows 92.5% sequence identity with AtMYB72.

A

```
BGLU42          -----MAQKLNLLNLAVP-----------------PVTHRSNFPSTFTFGVATSAYQIE
BGLU12          MGMGMAIAFLGVLALLVSNSWSAPTEPGSTILSNDVTLLNRSSFPAGFIFGTASSAYQYE
                         *.:* * *.                  .: :**.**: * **.*:**** *

BGLU42          GGWNEGKKGPSIWDKFTHIEG-KILDGSNGDVAVDHYHRYKEDVDLIGQLGFGAYRFSIS
BGLU12          GAANEGGRGPSIWDTLTHQHPEKVQDGSNGDVAVDQYHRYKEDVAIMKYMNTDAYRFSIS
                *. *** :******.:** .  *: **********:******** ::  :. .*******

BGLU42          WSRIFPDG-LGTEVNEEGIAFYNDLINTLLEKGIQPYVTLYHWDLPSHLQEAIGGWTNRK
BGLU12          WSRILPKGKISSGINQEGIKYYNNLINELIANGLEPFATLFHFDLPQALQDEYSGFYSPN
                ****:*.* :.: :*:*** :**:*** *: :*::*:.**:*:***. **:  .*: . :

BGLU42          IVDYFGLYADACFANFGDRVKHWITLNEPLQTSVNGHCIGIFAPGRNEKPLIEPYLVSHH
BGLU12          IIKDFEDYSELCFKEFGDRVKHWITLNEPWSYSVG---------------VDKPYLATHY
                *:. *  *:: ** :************** . **.               : :***.:*:

BGLU42          QVLAHATAVSIYRSKYKESQGGQIGLSVDCEWAEPNSEKPEDKVAADRRIDFQLGWFLDP
BGLU12          QLLAHAASVQIYKAKYQVSQKGLIGITLNSGWFLPYSNNTLDVHAAQRALDFAFGWFMEP
                *:****::*.**::**: ** * **::::. *  * *::. *  **:* :** :***::*

BGLU42          LFFGDYPASMRQKLGDNLPRFTPEEKEFMLQNSWDFLGLNHYTSRLISHVSNKEAESN-F
BGLU12          ITRGKYPRSMEKYVGSRLPNFTEEQSKQLIG-SFDFIGLNYYTSNYAAHLFHPNNDTDPT
                :  *.** **.: :*..**.** *:.: ::  *:**:***:***.  :*: : : :::

BGLU42          YQAQELERIVELENGDLIGERAASDWLYAVPWGIRKTLNYMSKKYNHPPIFITENGMDDE
BGLU12          YWTDQHVNLTSERNGIPIGPRAASSWLYVYPKGIEELLLYITKTYNNPLIYITENGIDEF
                * :::  .:.. .**  ** ****.***. * **.: * *::*.**:* *:*****:*:

BGLU42          DDGSASIHDMLDDKRRVDYFKSYLANVSQAIEDGVDIKGYFAWSLLDNFEWAQGYTKRFG
BGLU12          NDPTLSLEEALIDTFRIDYHYRHLYYVSTAIKDGANVKGYFAWSLLDNFEWFNGYTVRFG
                :* : *:.: * *. *:**.  :*  ** **:**.::************** :*** ***

BGLU42          LVYVDYKNGLTRHPKSSAYWFMKFLKGDEENKGKKE
BGLU12          INFVDYKDNLKRHQKLSGHWFRDFLKKN--------
                : :****:.*.** * *.:** .*** :
```

B

```
MYC2            MTDYRLQPTMNLWTTDDNASMMEAFMSSSDISTLWPPASTTTTTATTETTPTPAMEIPAQ
MYC4            ---------MNLWTDDN-SSVMEAFMTSSDLSSLWPPP------------PPPPQSASSA
                         ***** *: :*:*****:***:*:****.            *.*. . .:

MYC2            AGFNQETLQQRLQALIEGTHEGWTYAIFWQPSYDFSG---ASVLGWGDGYYKGEEDKANP
MYC4            AVFNQDTLQQRLQALIEGARESWTYAIFWQSSYDYPSSASTAVLGWGDGYYKGDEDKG--
                * ***:************::*.********.***:..   ::***********:***.

MYC2            RRRSSSPPFSTPADQEYRKKVLRELNSLISGGVAPSDDAVDEEVTDTEWFFLVSMTQSFA
MYC4            --KAKSTKTTTPAEQDHRKKVLRELNSLISGSAAAPSDDVEEEVTDTEWFFLVSMTQSFV
                  ::.*.  :***:*::**************..*...* *:*****************.

MYC2            CGAGLAGKAFATGNAVWVSGSDQLSGSGCERAKQGGVFGMHTIACIPSANGVVEVGSTEP
MYC4            SGTGLPGQAFYHSSPVWLTGPDRLAGSSCERARQGQVFGLQTLVCIPSSNGVVELGSTEM
                .*:**.*:**  ...**::*.*:*:**.****:** ***::*:.****:*****:****

MYC2            IRQSSDLINKVRILFNFDGG------AGDLSGLNWNLDPDQGENDPS-MWINDP------
MYC4            IFQNPDLMNKVRILFNFNSNSIDVGSSWPLTGSTTTTAADQGENDPSSLWLNDSEIRDSV
                * *..**:*********:..      :  *:* . .  .******** :*:**.

MYC2            ------IGTPGSNEPGNGAPSSSSQLFSKSIQFENGSSSTITENPNLDPTPSPVHSQTQN
MYC4            TTVTTVTTTTPASVISVNPGSSALAETPSSVHLPNNNNNTHAGGASQSQNRSFFSRELNF
                        *. :.  . .. **:    ..*::: *....* : ... . . * .  : :

MYC2            PKFNNTFSRELNFSTSSSTLVKPRSGEILNFGDE--------GKRSSGNPDPSSYSGQTQ
MYC4            SEFGFDGSNAVKTGNGQHHSLKPESGEILSFGESKRSSYGGGGGGGGGGNANFFSGQSQ
                .:*.   *. :: ....   :**.*****.**:.        *   ..*. :.. :***:*

MYC2            FENKRKRSMVLNEDKVLSFG----------------------DKTAGESDHSDLEASVV
MYC4            FVAAAEDNNGKKRKSPNSRGSNNDDGMLSFTSGVILPPSNMKSSGGGGDSDHSDLEASVV
                *    : .   :...  * *                       .  .*:***********

MYC2            KEVAVE------KRPKKRGRKPANGREEPLNHVEAERQRREKLNQRFYALRAVVPNVSKM
MYC4            KENESSRVVEPEKRPRKRGRKPANGREEPLNHVEAERQRREKLNQRFYALRAVVPNVSKM
                **    .     ***:*******************************************

MYC2            DKASLLGDAIAYINELKSKVVKTESEKLQIKNQLEEVKLELAGRKASASGG---------
MYC4            DKASLLGDAISYITELKSKLQTLESDKDGLEKQLDSLKKDLDNVKKEASSAPPPLPDKEL
                **********:**.*****: . **:*  :::**:.:* :* . * .**..

MYC2            --DMSSSCSSIKPVGMEIEVKIIGWDAMIRVESSKRNHPAARLMSALMDLELEVNHASMS
MYC4            RMSSNNLVGGGKLIDLDIDVKIIGWDAMIRIQCSKKNHPAARLMAALMELDLDVHHASVS
                  . ..  .. * :.::*:***********::.**:********:***:*:*:*:***:*

MYC2            VVNDLMIQQATVKMGFRIYTQEQLRASLISKIG----
MYC4            VVNDLMIQQATVKMGSRFYTQEQLRSALTSKVGGDVR
                *************** *:*******::* **:*
```

Figure S5: Sequence alignment showed that AtBGLU12 and BGLU42 of peanut had 44% identity **A,** and *AtMYC2* had 45.7% identity with peanut *MYC4* **B.**

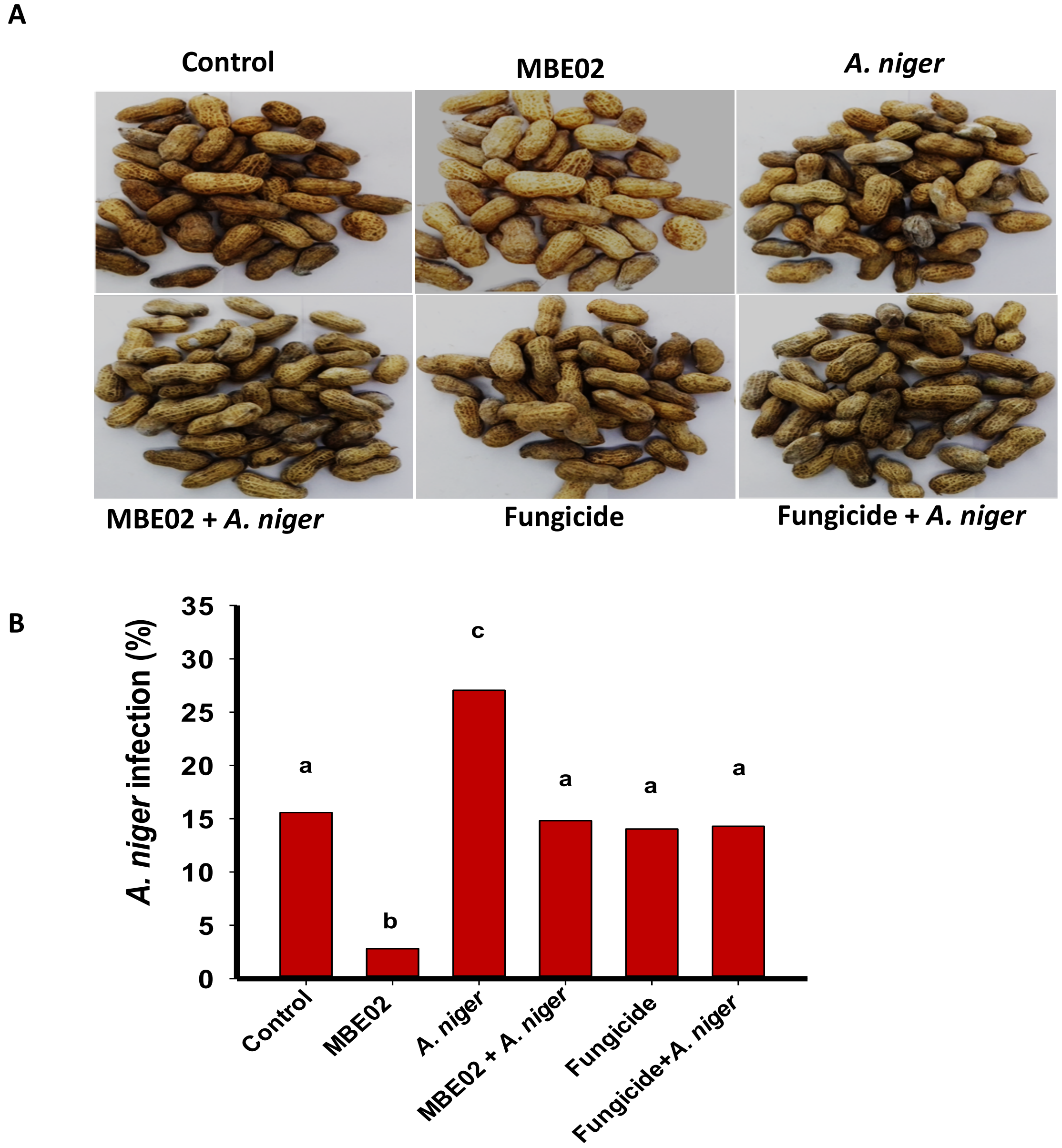

Figure S6: ***In vitro pod*** **colonization assay with** ***A. niger*** **in presence of MBE02**. **(a) and (b)** respectively show that peanut seeds were incubated with control (water only), MBE02 (O.D. 0.6), *A. niger* (10^6 spore/ml) and fungicide for 30 min. After incubation, half seeds from MBE02 and fungicide treatment were incubated with *A. niger* (10^6 spore/ml) for 30 min and kept in the dark for 72 h. Data are means ± SE (n=30-50). Picture of a representative plate is shown. Different letter shows statistical difference among the treatments calculated by SNK test ($p<0.05$).

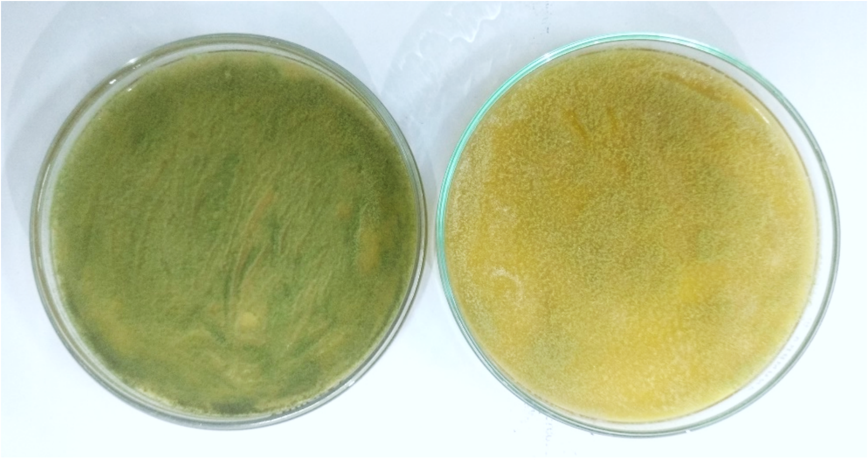

A. flavus **A. flavus + MBE02**

Figure S7. MBE02 alters the growth of *A. flavus*. Plate assay to show inhibitory effect of MBE02 on *A. flavus*. Spore suspension of fungi (10^6 spores/ml) were spreaded throughout the plate and inoculated with two spots of 5µl MBE02 (O.D. 0.6) followed by incubation at 25±2ºC. Picture was taken after 7 days of incubation.
