# Supplementary material for: A halotolerant growth promoting rhizobacteria triggers induced systemic resistance in plants and defends against fungal infection
Source: Sci Rep. 2019 Mar 11;9:4054. doi: 10.1038/s41598-019-40930-x (PMC6411892; doi:10.1038/s41598-019-40930-x)
Supplement: Supplementary file 5 — Table S4 [file 41598_2019_40930_MOESM5_ESM.pdf]

Table S4. Gene ontology enrichment for differentially expressed genes in MBE02 treatment than the control. GO analysis was performed with Blast2GO as described in the text.

Biological Process

GO_ID	GO term	Frequency	P-value	log10 p-value
GO:0016310	phosphorylation	7.764%	6.17163E-05	-4.2096
GO:0009058	biosynthetic process	31.611%	0.000829469	-3.0812
GO:0006355	regulation of transcription, DNA-templated	9.917%	0.00128736	-2.8903
GO:0006351	transcription, DNA-templated	10.659%	0.00128736	-2.8903
GO:0055114	oxidation-reduction process	15.060%	0.002156751	-2.6662
GO:0006952	defense response	0.568%	0.003274161	-2.4849

Cellular component

GO_ID	GO term	Frequency	P-value	log10 p-value
GO:0005634	nucleus	8.965%	0.00054325	-3.265
GO:0016020	membrane	61.592%	7.87046E-05	-4.104
GO:0016021	integral component of membrane	55.868%	0.030040005	-1.5223
GO:0005737	cytoplasm	26.017%	0.00054325	-3.265

Molecular function

GO_ID	GO term	Frequency	P-value	log10 p-value
GO:0004672	protein kinase activity	3.390%	6.17163E-05	-4.2096
GO:0000166	nucleotide binding	20.185%	6.17163E-05	-4.2096
GO:0016747	transferase activity, transferring acyl groups (	2.285%	0.000829469	-3.0812
GO:0003700	transcription factor activity, sequence-specifi	4.217%	0.00128736	-2.8903
GO:0003677	DNA binding	12.549%	0.00128736	-2.8903
GO:0046872	metal ion binding	15.425%	0.002156751	-2.6662
GO:0043531	ADP binding	0.183%	0.003274161	-2.4849
GO:0005215	transporter activity	8.494%	0.00414763	-2.3822
GO:0016758	transferase activity, transferring hexosyl grou	0.898%	0.030040005	-1.5223
GO:0016740	transferase activity	21.036%	0.031174536	-1.5062
